# Biofabrication of Electrospun Scaffolds for the Regeneration of Tendons and Ligaments

**DOI:** 10.3390/ma11101963

**Published:** 2018-10-12

**Authors:** Alberto Sensini, Luca Cristofolini

**Affiliations:** 1 Department of Industrial Engineering, School of Engineering and Architecture, Alma Mater Studiorum—Università di Bologna, 40131 Bologna, Italy; alberto.sensini2@unibo.it; 2 Health Sciences and Technologies—Interdepartmental Center for Industrial Research (HST-ICIR), Alma Mater Studiorum—Università di Bologna, 40064 Ozzano dell’Emilia, Bologna, Italy

**Keywords:** electrospinning, tendons and ligaments, scaffolds, biofabrication, hierarchical structure, non-linear mechanical properties

## Abstract

Tendon and ligament tissue regeneration and replacement are complex since scaffolds need to guarantee an adequate hierarchical structured morphology, and non-linear mechanical properties. Moreover, to guide the cells’ proliferation and tissue re-growth, scaffolds must provide a fibrous texture mimicking the typical of the arrangement of the collagen in the extracellular matrix of these tissues. Among the different techniques to produce scaffolds, electrospinning is one of the most promising, thanks to its ability to produce fibers of nanometric size. This manuscript aims to provide an overview to researchers approaching the field of repair and regeneration of tendons and ligaments. To clarify the general requirements of electrospun scaffolds, the first part of this manuscript presents a general overview concerning tendons’ and ligaments’ structure and mechanical properties. The different types of polymers, blends and particles most frequently used for tendon and ligament tissue engineering are summarized. Furthermore, the focus of the review is on describing the different possible electrospinning setups and processes to obtain different nanofibrous structures, such as mats, bundles, yarns and more complex hierarchical assemblies. Finally, an overview concerning how these technologies are exploited to produce electrospun scaffolds for tendon and ligament tissue applications is reported together with the main findings and outcomes.

## 1. Introduction

In the last three decades the topic of tissue regeneration is getting extreme attention in the orthopedic research field [1,2,3]. The use of scaffolds allows driving cells to proliferate and regenerate tissues in specific directions [4]. This property is fundamental to produce devices able to guide cells to the regeneration of the collagen anisotropy in the musculoskeletal tissues, such as tendons or ligaments [5,6,7]. In fact, due to the low vascularization, the hypocellularity, the anisotropy and the non-linear mechanical properties of these tissues, natural tendon and ligament regeneration is particularly complex [8]. Among the various techniques proposed in the literature to produce scaffolds, electrospinning, and its ability to produce nanofibers, is definitely one of the most promising for tendon and ligament tissue engineering. Several works are published annually on this topic, presenting electrospun scaffolds with increasingly improved biomimicry and enhanced cellular response [9,10,11]. 

The aim of this review is to analyze methods to build electrospun scaffolds for the regeneration of tendons and ligaments, and to illustrate the most promising applications. This review is conceived to give a general background of the main electrospinning setups to produce and collect nanofibers, and then to focus on the applications to produce scaffolds for tendon and ligament tissue engineering.

## 2. Methods of the Literature Search and Review

A systematic search using ScienceDirect, Scopus, PubMed and Google Scholar and Google Patents databases was performed until March–July 2018. Papers relevant to the electrospinning methodologies to produce scaffolds and their applications related to tendons and ligaments regeneration and replacement, published between 1990 and 31 July 2018, were selected:

- The following search string was used to retrieve the manuscripts presenting equipment and techniques to produce electrospun scaffolds: “electrospinning AND (review OR technique OR setup OR production process OR equipment OR methods OR scaffold production OR scaffold manufacturing)”.
- The following search string was used to retrieve the manuscripts presenting applications of electrospun scaffolds for regeneration and replacement of tendons and ligaments: “electrospinning AND (review OR regeneration OR repair OR tendon OR ligament OR bone OR muscle OR insertion)”.

Moreover, to find additional papers possibly missed through the database searches, the list of citations from every paper was scanned. The title, abstract and main text of each work were examined, and only the papers truly relevant for this review were cited and incorporated. Inclusion criteria were manuscripts in English reporting electrospun scaffolds designed for tendon and ligament tissue regeneration and replacement applications. 

The different electrospinning techniques listed in the Section 5 “Equipment and Techniques to Produce Electrospun Scaffolds” were divided into seven categories: mats of nanofibers and multilayered scaffolds, short and finite length bundles and yarns, continuous bundles, continuous yarns, tubes and conduits, textiles of nanofibers, multiscale hierarchical scaffolds. 

For each paper of the Section 6 “Applications for Tendon and Ligament Regeneration and Replacement,” the materials used, the specific application, manufacturing methods and principal outcomes of the work are summarized. The papers are divided into six main categories and six subcategories: preliminary studies on electrospun materials (flat electrospun mats, multilayered and co-electrospun scaffolds), patches and augmentation grafts (patches, augmentation grafts), multiscale hierarchical scaffolds for massive replacement (fascicle inspired bundles and yarns, hierarchically structured scaffolds), bone insertion, muscle insertion, and tendon and ligament healing and anti-adhesion. Finally, in vitro studies are reported for each category and subcategory, followed by the in vivo applications.

## 3. Tendons and Ligaments: Properties and Replacement

### 3.1. Morphological and Mechanical Properties of Tendons and Ligaments

To design scaffolds able to properly regenerate tendon and ligament tissues, it is mandatory to start by analyzing tendons and ligaments morphology and mechanical properties.

Despite the different physiological functions (connection and load transfer between a bone and a muscle (tendon) and between two bones (ligament)), and the different morphologies (depending also on the anatomical site), tendons and ligaments have similar composition and hierarchical structure [12,13]. 

Tendons and ligaments are filamentous collagen structures, composed approximately by an 80% of extracellular matrix. The remaining 20% are cells (fibroblasts or tenocytes), arranged in rows between the collagen fibers [14,15]. Approximately 70% of the total extracellular matrix is composed by water and the remaining 30% of solid material [15]. Collagen accounts for 70–80% of the dry weight of tendons and ligaments and Type I collagen accounts for 60–85% of the total collagen [12,14]. Type I collagen confers stiffness and strength to the tissue but other types of collagen exist in minor amounts, namely Type III, V, X, XI, XII and XIV collagens [12,14]. Type V collagen has been associated to Type I collagen in the regulation of the collagen fibril diameter while Type III collagen is functionalized in tendon repair. Type XII collagen is present in the surface of fibrils and bonds them with other matrix components such as decorin and fibromodulin [14]. The remaining part is composed by the ground substance (non-fibrous component of the matrix), basically comprised of hyaluronan, glycoproteins, and proteoglycans, and modulates tissue metabolism, provides shock absorption, decreases internal friction between collagen fibers, and binds water [15]. 

**Figure 1:**
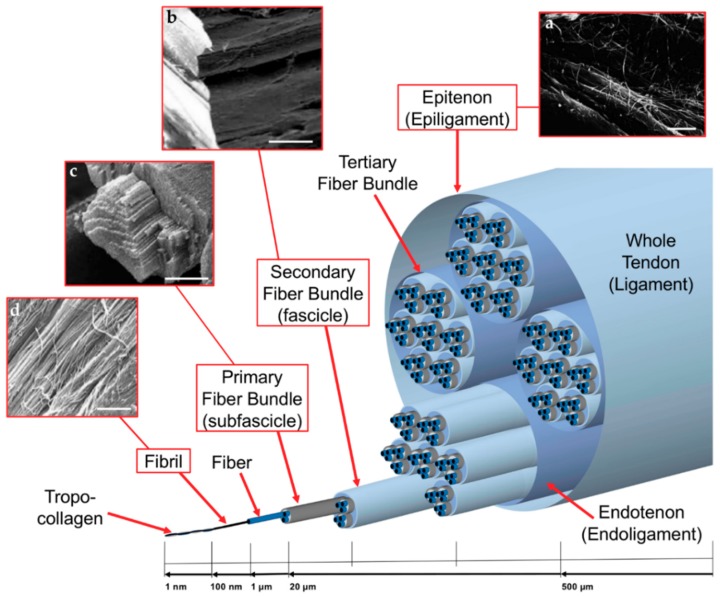
Hierarchical arrangement of the collagen of tendons and ligaments: (**a**) Scanning electron microscopy (SEM) of epitenon collagen fibrils (scale bar = 2 micrometers, adapted from Kannus et al. [6], reproduced with permission. Copyright 2008, John Wiley and Sons.); (**b**) SEM image of a collagen fascicle (scale bar = 100 micrometers); (**c**) SEM image of a collagen bundle (scale bar = 45 micrometers); and (**d**) SEM image of collagen fibrils (scale bar = 1.8 micrometers). (**b**–**d**) SEM images adapted from Moshiri et al. [17], reproduced with permission under the terms of the CC BY 4.0 license. Copyright 2013, OMICS Publishing Group.

Morphologically speaking, tendons and ligaments are composed of a complex hierarchical structure of collagen fibrils, axially aligned with the tendon/ligament and connected to each other in different levels of aggregation (Figure 1). There is no standard nomenclature for aggregations of collagen fibrils within the tendon, perhaps due to their great variability depending on the function and the anatomical site [6]. The basic unit of tendons and ligaments is tropocollagen molecule, which is a long, thin protein produced inside a cell (e.g., fibroblast) and secreted into extracellular matrix as procollagen [16]. Tropocollagen molecules aggregation produce the collagen fibril, which is the smallest structural unit of the tendon and ligament tissue. The diameter of the fibrils ranges 10–500 nm, depending on species, age, and anatomical location [16]. A bunch of collagen fibrils forms a collagen fiber [6]. A bunch of collagen fibers forms a primary fiber bundle (sub-fascicle), and a group of primary fiber bundles forms a secondary fiber bundle (fascicle). A group of secondary fascicles, in turn, forms a tertiary bundle, and the tertiary bundles make up the tendon, which is surrounded by the epitenon/epiligament [6]. The structures from fibers to tertiary fiber bundles are surrounded by a thin collagen membrane called endotenon/endoligament, containing blood vessels, lymphatics and nerves [5,6,16].

**Figure 2:**
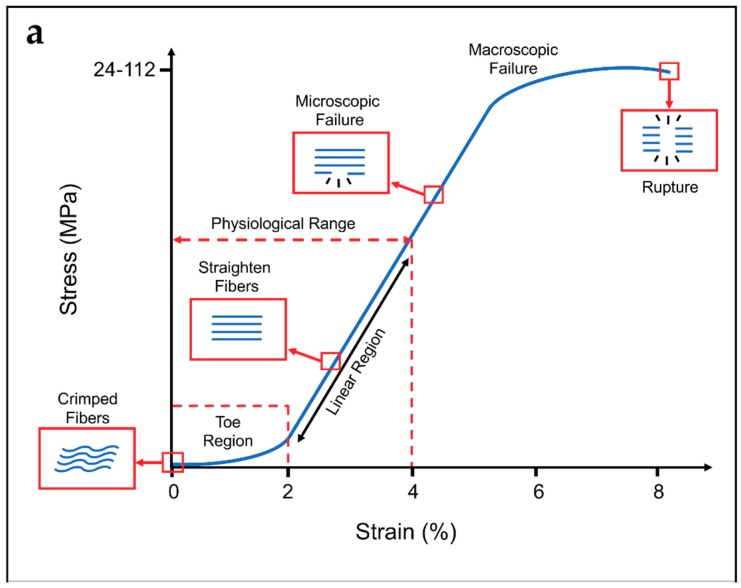

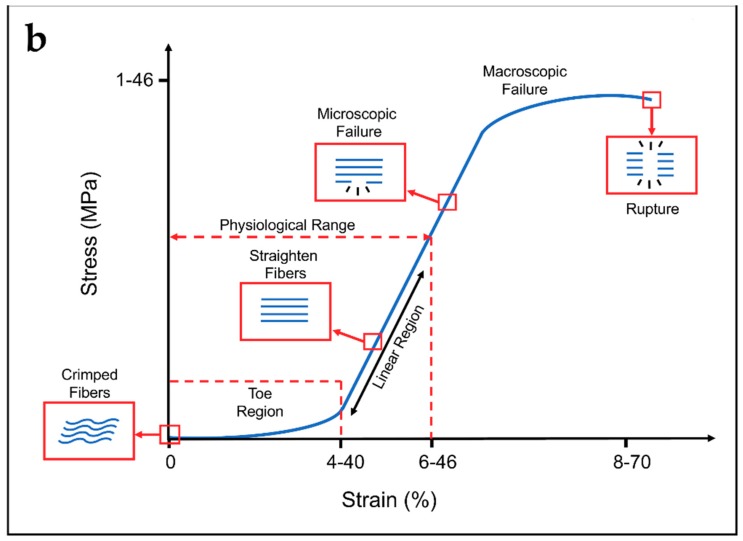
Typical stress–strain curve and schematization of the behavior of the collagen fibers for: (**a**) tendons; and (**b**) ligaments. Typical ranges of stress and strain are indicated on the x and y axes.

This rope-like hierarchical structure confers to tendons and ligaments typical non-linear mechanical properties (Figure 2) [7,18,19]. When a load is applied to a tendon or ligament, the collagen fibers, which are crimped at rest (crimping angle depending on different tendons/ligaments), start to align with each other losing the crimped behavior until 2% of strain (toe region). Due to the load transfer function of the tendons, the toe region of their stress–strain plot is quite short (2–5%), and similar in each tendon of the human body. Conversely, ligaments must allow different range of motions in different joints, and consequently show wider ranges of strain for the toe region depending on the different anatomical sites (anterior cruciate ligament: 4%; spine ligaments: 10–40% [15,20,21,22]). In the linear region of the stress–strain plots, the fibers of collagen are straightened and provide a quite ideal elastic recovery, if load is removed. After the linear region, the fibers progressively start sliding with respect to each other. This event is followed by progressive failure of the fibers, until complete failure of the tendon or ligament [14,16,18,19]. The mechanical properties are strongly related to the cross-section and function of the particular tendon or ligament of the body and from the strain rate with which the load is applied [7,15]. For example, the range of failure stresses may vary in tendons from the 24–69 MPa of the patellar to the 112 MPa of the gracilis, and in ligaments from the 1–15 MPa of the flavum to the 24–46 MPa of the lateral collateral [15]. Moreover, the nature of the weak bonds of collagen and the presence of water are responsible for the viscoelasticity of tendons and ligaments [7,15].

Considering these common morphological and mechanical characteristics, scaffolds for tendon and ligament tissue regeneration are often quite similar in their structure and properties.

### 3.2. Generic Requirements of Scaffolds for Tendon and Ligament Regeneration

Considering the morphology and the biomechanical properties of tendons and ligament previously described, the general requirements for a scaffold for the regeneration of these tissues are listed below [23,24]:

1. Biocompatibility: Scaffolds must be biocompatible and made of natural or synthetic materials. This encourages the cells to grow, infiltrate and proliferate on and into the scaffolds, reproducing the physiological collagen. Biocompatibility is also fundamental to prevent and minimize inflammatory phenomena which could compromise the regenerative process [17,23,24,25].
2. Biodegradability: Scaffolds need to be progressively degraded by cells and body fluids. Therefore, they must be properly engineered to permit that the degradation rate could allow cells to reproduce the natural collagen without being resorbed too fast. Moreover, the products of the degradation must not produce any inflammatory or toxic effects to cells and their surrounding tissues [17,23,24,25].
3. Mechanical Properties: To permit a correct replacement of the site of the lesion, cells have to feel the physiological stiffness of the substrate, and experience physiological levels of strain, and also have to be stimulated to reproduce collagen and proliferate [17,25,26]. For these reasons, the scaffolds need to be designed to provide mechanical properties in the range of the specific tendon or ligament. However, to prevent damages of the surrounding tissues after the suture in the site of the lesion, scaffolds must be less stiff and less strong compared with the host tendon or ligament. Finally, a degree of ductility before the nominal failure load is required to prevent an unexpected and abrupt failure of the scaffolds in case of an overload.
4. Morphology: Tendons and ligaments are composed of nanometric and axially aligned collagen fibrils connected in different hierarchical levels. A scaffold designed for tendon and ligament tissue regeneration needs to be produced with the same philosophy. In fact, fiber-like scaffolds permit cells to grow, attach and reproduce the collagen following the direction of alignment of the fibers, contributing to confer tendons’ and ligaments’ morphology and mechanical properties to the regenerated tissue.
5. Porosity: Scaffolds also need to be porous to allow the cells’ infiltration [17,24,25,26]. Interconnected networks of porosities are essential for cell nutrition, proliferation, and migration for tissue vascularization and formation of new tissues [27,28]. A porous network structure assists in guiding and promoting new tissue formation [29,30]. Materials with high porosity allow releasing biofactors such as proteins and genes, providing good substrates for nutrient exchange between the cells [31].

For these reasons, among the various techniques to regenerate and replace tendons and ligaments [14,26,32], electrospinning is one of the most promising techniques to develop scaffolds for tendon and ligament tissue engineering.

## 4. The Electrospinning Technique: An Introduction

### 4.1. Electrospinning Operating Principles 

**Figure 3:**
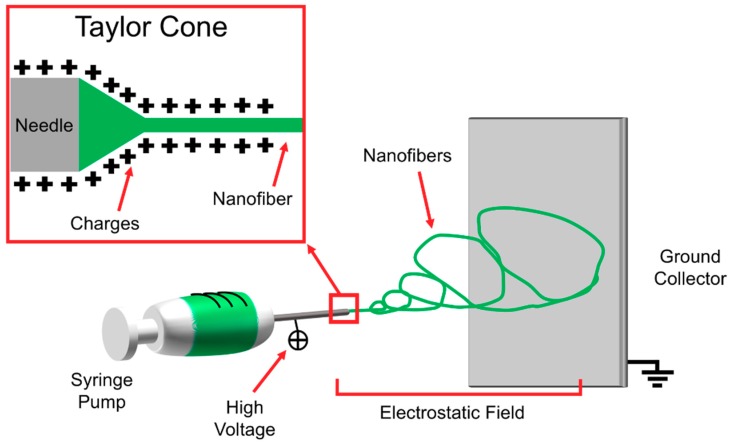
Electrospinning operating principle and Taylor cone formation.

Electrospinning technology is an electrically-driven method to produce fibers of nanometric or micrometric diameter. Electrospinning was invented in the early twentieth century [33], and it has attracted a lot of attention in the last three decades in the field of tissue engineering, thanks to its ability to mimic the extracellular matrix [34,35,36,37]. The formation of nanofibers through electrospinning is based on the uniaxial stretching of a viscoelastic solution [38]. The process requires only a few elements: a syringe charged with a polymeric solution provided with a metallic needle, a syringe pump, a high voltage power supply and a collector, generally at ground potential (Figure 3). In the electrospinning process, a high voltage is used to create an electrically charged jet of polymer micro- or nanofibers by the syringe [39]. When the solution is slowly pumped out of the needle tip through the spinneret, it forms a spherical droplet driven by surface tension. As the droplet is connected to the high voltage power supply, its surface will be quickly covered by charges of the same sign. The repulsion among these charges destabilizes the spherical shape. When the repulsion is strong enough to overcome the surface tension, the droplet deforms into a conical shape (called Taylor cone), and a jet will emanate from the apex of the cone [40]. The repulsive forces, generated by the charges on the surface of the fibers, cause the whipping of the liquid jet towards the collector. This whipping motion induces the polymer chains inside of the solution to stretch and slide past each other. The result of this process consists in the creation of fibers with small enough diameters to be called nanofibers [41]. The distance between needle and collector is important to determine the morphology of the electrospun nanofiber, and depends on the polymeric solution. In general, large-diameter nanofibers are formed when the distance is small, whereas the diameter decreases as the distance is increased [41,42,43,44]. The solvent system plays a key role to prevent the formation of undesirable beads. Highly volatile solvents must be avoided because their low boiling points and high evaporation rates cause the drying of the jet at the needle tip, blocking the electrospinning process. Less volatile solvents must be avoided too, because their high boiling points prevents suitable drying of the nanofiber jet flight. The deposition of solvent-containing nanofibers on the collector will cause the formation of beaded nanofibers [45,46]. The conductivity and dipole moment of the solvents are also important [41,47].

The electrospinning process is strongly dependent on three families of parameters [41]:

1. Solution parameters: Polymers and solvents, viscosity, conductivity of solvents and polymers, and concentration of the polymers.
2. Electrospinning parameters: Flow rate of the pump, diameter and shape of the needle, applied voltage, distance between the needle and the collector, and shape and movement of the collector.
3. Environmental parameters: Temperature and relative humidity.

Tuning properly the combination of all these parameters, it is possible to tune the final morphology, cross-section and orientation of the nanofibers produced [38,39,40,41,48]. Due to its ability to produce nanofibers, even made of resorbable materials, with a morphology similar to the one of collagen fibrils of tendons and ligaments, electrospinning is very promising for the regeneration and replacement of these tissues [9,10]. Electrospinning is also suitable to produce scaffolds that are able to reproduce the typical non-linear toe region and the biomechanical properties of tendons and ligaments [10].

### 4.2. Materials for Tendon and Ligament Tissue Regeneration 

The use of suitable materials is fundamental for scaffolds that have to regenerate and replace tendon and ligament tissue. A wide range of both resorbable and non-resorbable polymers, to produce electrospun nano- and microfibers for tendon and ligament applications, can be found [9,10,14]. In particular, natural or synthetic bioresorbable materials are indicated for young patients or sport athletes due to their faster cellular and metabolic activity [49]. Conversely, non-resorbable (inert) materials are preferred for tendon and ligament replacement in elderly patients, because of their lower cellular activity and metabolic responses [26]. In fact, it is well established that the mechanical properties of tendons and ligaments decrease according to the age of the patients and tend to become stiffer [50,51,52].

A wide range of natural or synthetic biopolymers is investigated for tendon and ligament tissue regeneration and replacement applications by means of electrospinning (Table 1). In some cases, nanofibers are also electrospun from polymers blends, or in a core–shell configuration (Table 2). Finally, even the possibility of loading nanofibers with different natural or synthetic nanoparticles or drugs is widely exploited (Table 3).

## 5. Equipment and Techniques to Produce Electrospun Scaffolds for Tendon and Ligament

The complex multiscale structure composing tendons and ligaments suggests researchers investigate different electrospinning setups for mimicking these kind of tissues as closely as possible [5,6,7]. Some of these setups, however, were applied in other research fields before being used for tissue engineering [9,132,133,134]. In this section, the most common setups and configurations are presented to produce electrospun nanofibers for tendon and ligament tissue regeneration.

### 5.1. Mats of Nanofibers and Multilayered Scaffolds 

**Figure 4:**
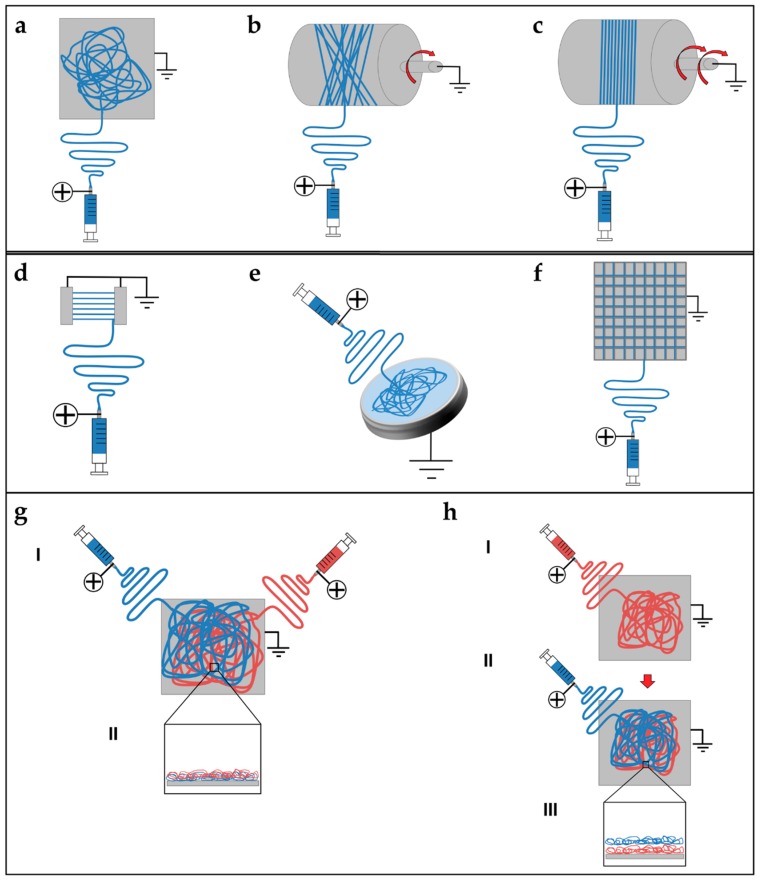
Different electrospinning setups to produce mats of nanofibers: (**a**) flat plate collector; (**b**) drum collector rotating at low speed, producing random nanofibers; (**c**) drum collector rotating at high speed, producing aligned nanofibers; (**d**) gap collector; (**e**) liquid bath collector; (**f**) flat plate collector with a grid pattern on the surface; (**g**) mixing electrospinning or co-electrospinning setup ((I) two syringes electrospin synchronously the solutions on a flat plate collector; and (II) section of the mat with the two different nanofibers mixed together; and (**h**) multilayering electrospinning setup ((I) one solution electrospins on a flat plate collector producing a random mat; (II) a second solution electrospins the previous random mat; and (III) section of the final mat shows two different layers of nanofibers overlapped).

Electrospun nanofibers are generally collected as nonwoven or randomly arranged structures, due to the “whipping instability” of the electrospinning jet [133]. By electrospinning on a flat ground plate (Figure 4a) or on a drum collector rotating at low speed (peripheral speed lower than 8 m/s) (Figure 4b), it is possible to obtain mats made by nanofibers with a random configuration [132,133,134]. Early studies concerning fiber deposition and assembly focus on controlling the fibers’ alignment. Aligned nanofibers can be collected by using a dynamic mechanical collector such as a cylindrical drum [135,136,137]. When the collector rotates at a high peripheral speed, on the order of ≥8 m/s, fibers start to align circumferentially (Figure 4c) [133]. It is also possible to align nanofibers by using two split parallel flat plates, also called gap collectors, connected with grounded electrodes (Figure 4d) [136,137,138,139,140,141,142,143,144,145,146]. This final collector setup guarantees a high alignment of the nanofibers but is very limited in the final length and thickness of the mat and fibers [9,132,133,134]. 

Replacing a classical metal collector with a liquid bath to collect nanofibers is the operating principle of the so-called wet electrospinning (Figure 4e). This setup is particularly suitable to increase the pore sizes of the electrospun mats, and remove the residual charge on their surface [147]. Ki et al. and Yokoyama et al. proposed preliminary works to produce mats of nanofibers by electrospinning on liquid baths of methanol and a blend of water and tertiary-butyl alcohol [148,149]. 

Another method to produce mats of nanofibers, to guide the deposition of the electrospun nanofibers in specific directions, relies on the deposition of specific patterns, for example lines or grids, on the flat plate collector [150] (Figure 4f). Zhang et al. proposed different patterns on different metal collector setups producing nanofibrous mats with different patterns organizations [151]. More recently, Nedjari et al. studied different nanofibrous mats obtained by metal flat plate collectors with different superficial patterns on them [152]. 

Mats nanofibers also present some morphological limitations because, using these techniques, only the planar configuration is possible. The mechanical properties of the electrospun mats increase from a random to an aligned configuration. However, mats of aligned nanofibers are not suitable to provide adequate yield and failure stress. 

Different techniques have been proposed to increase the mechanical properties of the nanofibrous mats, and also to electrospin different materials in the same structure. Kidoaki et al. developed two new different approaches for electrospinning two polymers together. The first is mixing electrospinning (named co-electrospinning), in which two different polymers are simultaneously electrospun from different syringes under special conditions. The polymer fibers are mixed on the same target collector, resulting in the formation of a mixed fiber mesh [153] (Figure 4g). The second is multilayering electrospinning that consists of electrospinning layer by layer different polymers on the same collector [153] (Figure 4h).

### 5.2. Short and Finite Length Bundles and Yarns 

**Figure 5:**
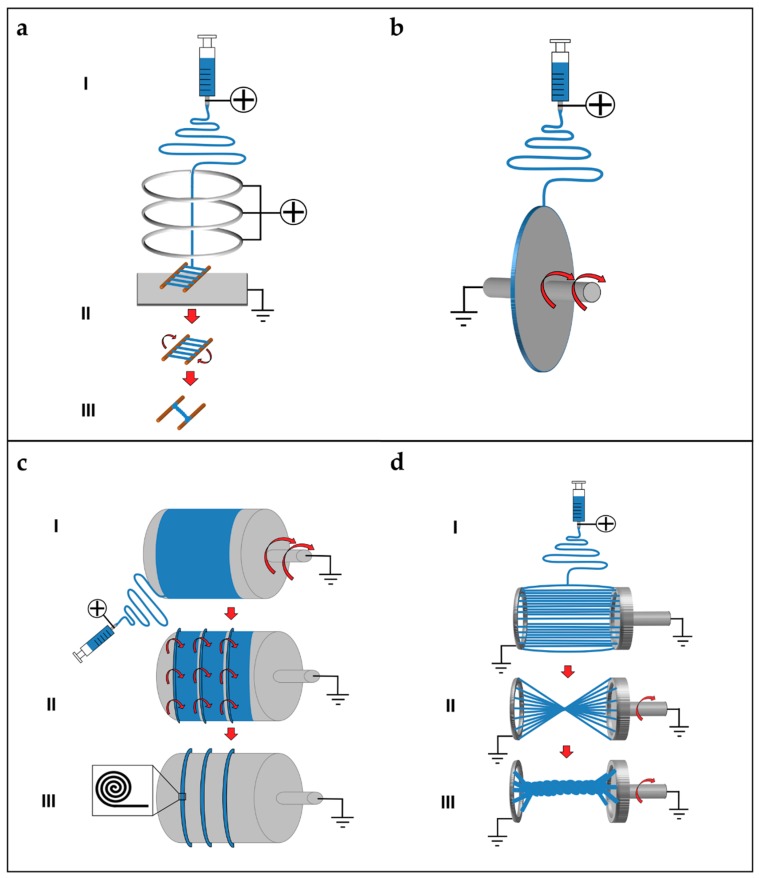
Different electrospinning setups to produce short yarns or bundles of nanofibers. (**a**) Short yarns production by three collimators rings: (I) the nanofibers travel through three metallic rings charged with the same polarity of the needle; (II) the nanofibers are aligned on two parallel wooden rods placed between the last ring and a flat plate ground collector; and (III) the mat of aligned nanofibers is manually twisted to obtain a short yarn [154,155]. (**b**) Tapered edge disk collector to produce short bundles of nanofibers [156]. (**c**) Finite length bundles production setup: (I) Nanofibers electrospin on a high-speed rotating drum collector to obtain aligned nanofibers; (II) the mat cut in strips which are manually wrapped on the drum; and (III) final bundles on the drum before being cut (box: schematic of a cross-section of a bundle) [59,60,93]. (**d**) Finite length yarns production with small discs setup: (I) solution electrospins on two parallel small metallic ground discs to obtain aligned nanofibers; (II) after the electrospinning session, one of the discs is put in rotation to twist the nanofibers; and (III) final yarn produced between the discs [162].

To overcome the mechanical limitations of the electrospun mats, alternative electrospinning configurations have been developed to obtain bundles and yarns of nanofibers. A bundle is a filament composed of aligned electrospun nanofibers. A yarn is a filament of twisted electrospun nanofibers [9,132,133,134]. The firsts concepts and patents on electrospinning bundles and yarns were proposed by Formhals [9,133,143,144,145,146]. Deitzel et al. used a series of rings, charged as the polymeric solution and a flat plate collector connected to the ground, to align nanofibers on two wooden rods, passed between the rings and the collector. After a thin mat of aligned nanofibers was formed between the rods, the mat was twisted in a short yarn (Figure 5a) [154,155]. Later, Theron et al. used a rotating disk collector, with a tapered edge, to obtain short nanofibrous bundles of PEO (Figure 5b) [156]. Further studies on this technique show that the fibers are prone to necking due to the high rotational speed of the collector. This necking effect severely reduces the material strength [9,157]. Short yarns are produced by twisting groups of nanofibers or mats. Fennessey et al. produced mats of aligned electrospun nanofibers of PAN, using a rotating drum collector. Mats were cut in tows, linked together and then twisted with an electric spinner to obtain yarns [133,158]. Lui et al. used a high speed rotating annular collector, to obtain circular aligned nanofibers membranes, which were cut and twisted to obtain yarns [159]. Uddin et al. divided into continuous strips mats of aligned nanofibers of PAN, reinforced with carbon nanotubes (CNTs), which were obtained by a rotating drum collector. After the production of the strips, they were twisted to obtain yarns [132,133,160]. More recently, Pauly et al. and Sensini et al. developed a technique where mats of aligned nanofibers, electrospun on a high-speed rotating drum collector, were cut in strips and manually wrapped to obtain bundles (Figure 5c). As a result, the length of the bundles produced with this method could be adjusted by changing the diameter of the collector [59,60,93]. 

Some groups designed automated methods to twist bundles and used some collector setups, modified from the gap collector shape, to obtain short bundles and yarns. Teo et al. produced bundles of aligned nanofibers using two close blade collectors, and then put into a water bath to compact the fibers [161]. A limitation of this approach is that, by increasing the distance between the collectors over 80 mm, a progressive reduction of deposition on the blades was observed [161]. Dalton et al. used two small discs as collectors: one could rotate and twist the aligned nanofibers to obtain a yarn (Figure 5d) [162]. Lui et al. produced yarns using a modified gap collector setup, based on the rotating ring collector and a rotating rod [159,163]. Later, Lotus et al. adopted the same philosophy of the modified gap collector proposed by Lui et al., but, by tuning the interaction between a rotating hollow hemisphere and a translating tapered metal rod, short nanofibrous yarns were obtained [163,164,165]. 

### 5.3. Continuous Bundles.

**Figure 6:**
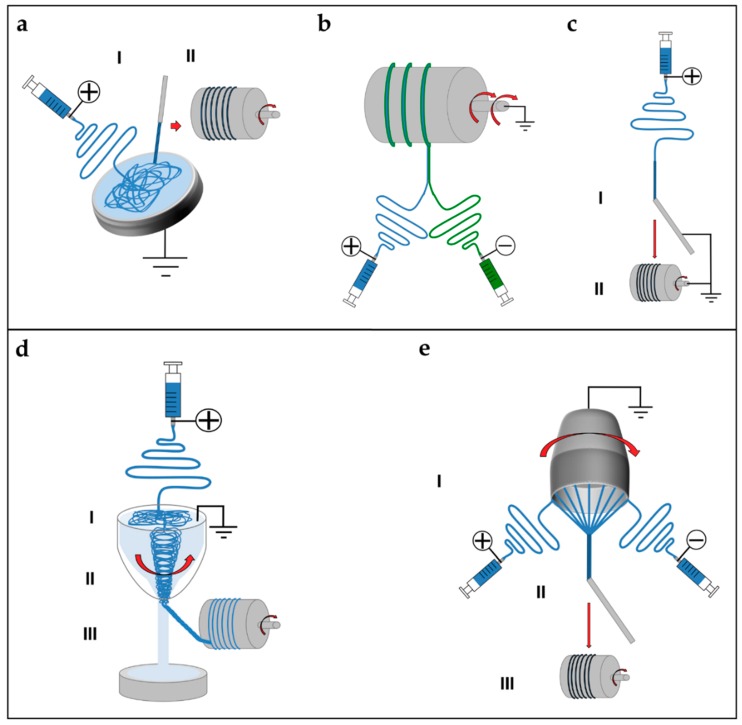
Different electrospinning setups to produce continuous bundles or yarns of nanofibers. (**a**) Continuous bundles production setup by a liquid bath collector: (I) solution electrospins on a liquid bath collector; and (II) nanofibers are taken by a glass rod and collected on a continuous rotating drum. (**b**) Continuous bundles obtained by co-electrospinning different solutions (oppositely charged) on a high-speed rotating drum collector. Nanofibers are attracted to each other in air. (**c**) Continuous bundles obtained by a needle: (I) a solution is electrospun starting by a needle connected to the ground; and (II) the starting bundle is guided to a rotating drum collector producing a continuous bundle. (**d**) Continuous yarns production with liquid bath vortex setup: (I) solution electrospins on a liquid bath collector; (II) nanofibers are twisted by a liquid vortex producing a yarn; and (III) the yarns pass through a hole in the bottom of the bath and collected by a drum collector [173,174]. (**e**) Continuous yarn production with funnel collector setup: (I) two syringes, opposite charges, electrospin nanofibers on the mouth of the funnel collector, in rotation, producing a mat; (II) a glass rod bring the center of the mat producing the yarn; and (III) the yarn is collected on a rotating drum [178].

While the methods described above can produce bundles/yarns of pre-defined length, other setups are developed to produce continuous and automatized bundles/yarns of nano- and microfibers. Smith et al., Kim et al. and Kim and Park utilized a setup, in which the polymeric solution was electrospun on a liquid collector with a high surface tension (distilled water or a blend with methanol) and collected on a rotating drum outside the solution (Figure 6a) [9,132,133,166,167,168]. Pan et al. developed a technique to produce bundles by co-electrospinning two polymers. Bundles are obtained by the attachment and alignment of electrospun nanofibers with opposite charges, in the air gap between the spinnerets and the drum collector (Figure 6b) [169]. Wang et al. produced continuous bundles, driving the alignment of the nanofibers, with a grounded needle and a rotating drum collector (Figure 6c) [170,171].

### 5.4. Continuous Yarns

The first example of continuous electrospun yarn was presented by Ko et al. who electrospun poly(lactic acid) (PLA) and PAN filled with carbon nanotubes, imposing first a twisting degree and, then, collecting the yarn on a rotating drum [172]. Teo et al. demonstrated the feasibility of producing continuous electrospun bundles and yarns with a water vortex (Figure 6d) [173,174]. Dabirian et al., using a static negative charged plate and a static rod, obtained a triangular jet of nanofibers. This configuration allows producing a yarn helped by the collecting system: a rotating drum, fixed on a rotating disk. The combination of the two rotations both permits the winding of the yarn and the modulation of its twisting degree [175]. Later, Dabirian et al. developed another setup to produce yarns, based on oppositely charged syringes posed on both sides of a charged collector. The nanofibers produced were then collected and twisted by the same rotating system [176]. Afifi et al. designed an electrospinning setup described for continuous aligning yarns. It comprises a slowly rotating grounded “funnel” target and a winder placed next to the funnel. A charged polymer jet was ejected from a needle. The electrospun fibers were first accumulated on the opening of the funnel to form a web. The web was then pulled upward and guided to a winder on which twisted fibers were continuously wound as a yarn [177]. Recently, Ali et al. developed a simple method to produce yarns. A rotating funnel was used to collect the nanofibers by two syringes opposite charged, on the edges of the funnel opening (Figure 6e) [178].

### 5.5. Tubes and Conduits

**Figure 7:**
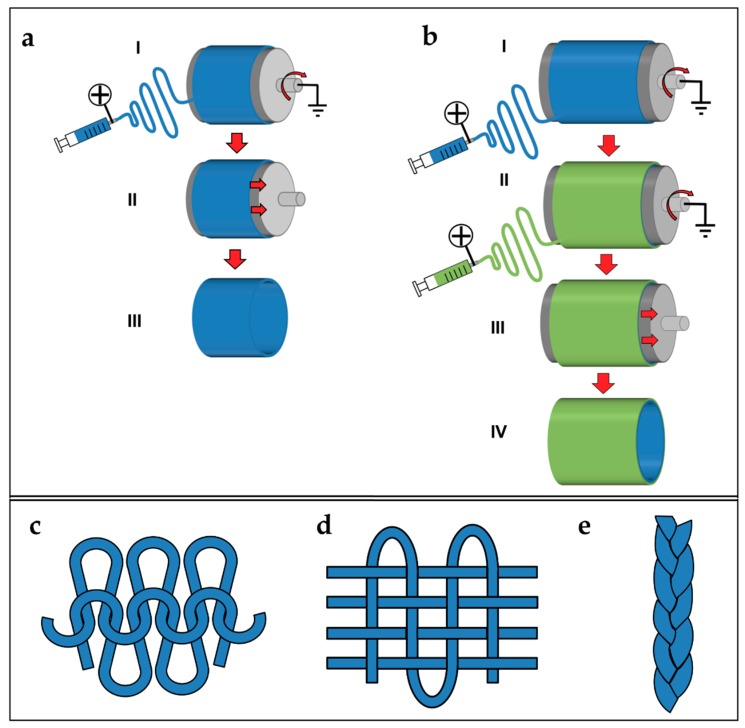
Different electrospinning setups and procedures to obtain tubes or conduits of nanofibers. (**a**) Tube of nanofibers obtained by a drum collector: (I) nanofibers are electrospun on a drum collector (by modulating the rotational speed, the nanofibers could be random or aligned); (II) the tube of nanofibers is removed from the drum; and (III) final tube of nanofibers. (**b**) Tube of different layers of nanofibers obtained by a drum collector: (I) the nanofibers of the first solution are electrospun on a drum collector (by modulating the rotational speed, the nanofibers could be random or aligned); (II) the nanofibers of the second solution are electrospun over the firs mat; (III) the mat of nanofibers is removed from the drum without cutting its side; and (IV) final tube of nanofibers with different nanofibers inside and outside. Different textile patterns to unit electrospun bundles or yarns of nanofibers: (**c**) Knitted; (**d**) Woven; and (**e**) Braided.

By tuning the diameter of the drum collector, and pulling it off from the mat, at the end of the electrospinning process, it is possible to obtain tubular conduits (Figure 7a) [38,179]. This kind of configuration is widely applied in tissue engineering and especially in the production of vessels and nerve conduits [38,179,180,181]. Firsts examples of nanofibers conduits in nerve tissue engineering were obtained by Bini et al., who collected random nanofibers on a rotating Teflon mandrel placed above a negative collector grid [182]. In the field of research of vascular tissue engineering, Stitzel et al. developed a random electrospun nanofibrous conduit, on a slowly rotating drum collector [183]. Matsuda et al. studied the disposition of the nanofibers on conduits, electrospinning segmented poly(urethane) (SPU) on a rotating drum collector with the speeds of 150 and 3400 rpm. At 150 rpm, the nanofibers are random, on both the internal and the external sides of the conduits. However, at 3400 rpm, the nanofibers are aligned on the internal side and random on the external side [184]. Vaz et al. and Kidoaki et al. upgraded the production of tubes and conduits, co-electrospinning different polymers in different layers of the same conduit (Figure 7b) [38,153,185]. 

### 5.6. Textiles of Nanofibers

To increase the mechanical properties of electrospun bundles and yarns and to unite these structures, different methods, derived from textiles production, are proposed. The methods most frequently used are knitting, waving and braiding [186]. Knitting is a well-established textile method to create complex two- and three-dimensional porous structures, from bundles or yarns that are interlaced in a highly ordered arrangement of connected loops. In the knitting process, bundles or yarns are drawn to form interconnected loops (Figure 7c) [186]. In tissue engineering, knitting structures made of fibers obtained by different techniques, have found applications, for example in cartilage, skin ligaments/tendons and blood vessels [187,188,189]. Weaving is a textile technique where two distinct sets of warps or wefts are interlaced at right angles to form a fabric with controlled strength, porosity, morphology, and geometry. Woven structures are lightweight, strong, and flexible (Figure 7d) [187]. Moutos et al. proposed several works about woven scaffolds for cartilage tissue engineering [190,191,192,193]. Finally, braiding scaffolds are often applied to unite groups of bundles or yarns, reducing the diameter of the final scaffold and increasing their mechanical properties. In braiding, complex structures or patterns are formed by inter-twining three or more fiber strands, which allows making cylinders and rods suitable for engineering connective tissues (Figure 7e) [187]. A wide variety of three-dimensional geometrical shapes with fine-tuned stable properties can be obtained through varying the arrangements of diagonally intertwined strands [186]. For instance, Wu et al. produced woven, knitted and braided scaffolds starting by their electrospun nanofibrous yarns finding interesting results about cells proliferation and mechanical properties [194].

### 5.7. Multiscale Hierarchical Scaffolds 

**Figure 8:**
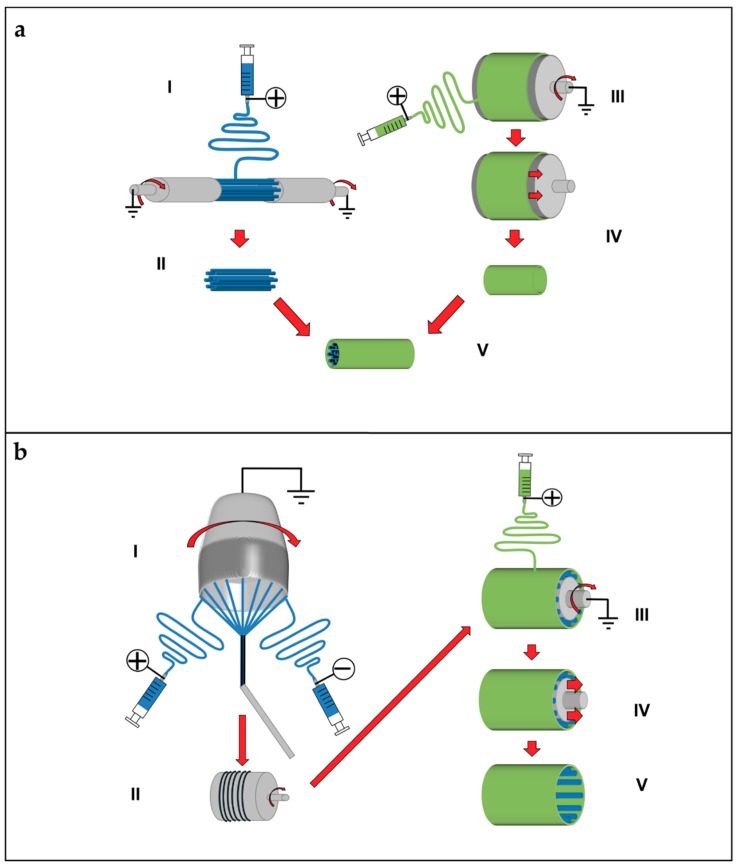
Different electrospinning procedures to obtain hierarchical multiscale scaffolds. (**a**) Nanofibers rod and conduit assembled in two steps: (I) nanofibers are electrospun on a rotating air gap collector; (II) the nanofibers rod is removed from the collector; (III) on a rotating drum collector, a random mat is electrospun; (IV) the mat is removed from the drum obtaining a conduit; and (V) the conduit is filled with the nanofibers rod [195]. (**b**) Yarns covered with a nanofibrous sheath: (I) continuous yarn of nanofibers produced with a funnel setup; (II) the continuous yarn is collected on a rotating drum; (III) the continuous yarn is cut in multiple yarns, fixed on rotating drum collector and covered with a nanofibrous sheath; (IV) the drum is removed from the scaffold; and (V) final scaffold [178,197].

The structures presented so far can be used to build hierarchically organized super-structures. Some groups tried to extend the concept of electrospun nanofibrous conduits, filling their central hollows with nanofibers or bundles/yarns of nanofibers. Li et al. submitted a patent for producing different configurations of scaffolds, by an electrospinning setup inspired from the principle of the gap collector. Two rotating cylinders allowed producing aligned nanofibers between them. The rod of aligned nanofibers is inserted in a nanofibrous conduit. The random nanofibrous conduit is produced by electrospinning on a drum collector and removed from it. Finally, the conduit is filled with the rod of aligned nanofibers (Figure 8a) [195]. Koh et al. assembled a nanofibrous scaffold for nerve regeneration matching conduits and bundles. First, a double layer conduit, with the inner part of aligned nanofibers and the external of random ones, is obtained by electrospinning the solution on a drum collector, rotating at different speeds. Then, the mat is axially cut and removed from the drum. The mat is rolled around a rod producing a conduit. After the removal of the nanofibrous conduit from the rod, the conduits central hollow is filled with nanofibers bundles of and nerve growth factor [173,196]. Recently, Li et al. presented a method for cover electrospun nanofibrous bundles with a random electrospun sheath, using a rotating drum collector. First, continuous bundles are produced. The bundles are cut in pieces and fixed on a drum collector rotating at low speed. Then, the nanofibers are electrospun on the bundles obtaining the final scaffold. Finally, the scaffold is removed from the drum collector, by sliding out the scaffold by the drum (Figure 8b) [178,197]. 

## 6. Applications for Tendon and Ligament Regeneration and Replacement

The previous section summarizes the most suitable electrospinning setups and methodologies to produce nanofibrous structures and scaffolds. In the present section, an overview of their applications in tendon and ligament tissue engineering is provided. The studies are divided according to the different clinical applications and the specific issues being solved. 

### 6.1. Preliminary Studies on Electrospun Materials

To start a preliminary investigation on the interactions between the cellular component and the electrospun nanofibers, the most suitable approach is on mats of random or aligned nanofibers. This is also useful to set the electrospinning parameters to obtain the desired morphology and arrangement of the fibers. 

#### 6.1.1. Flat Electrospun Mats

Lee et al. stimulated human ligament fibroblasts on electrospun mats of PU random and aligned nanofibers, with cyclic loads in a bioreactor. After seven days of dynamic culture, they found a statistically significant increment of the production of collagen Type I in the aligned mats compared to the random ones. They also found that fibroblasts were aligned in the direction of the aligned nanofibers [116]. In different studies for anterior cruciate ligament applications, Bashur et al. evaluated how the diameter and the orientation of electrospun PDLLGA and PEUUR fibers contributed to the morphology, orientation, and proliferation of NIH 3T3 fibroblasts and bone marrow stromal cells. Despite the growing capacity of fibroblasts on the different fiber meshes, they found that the aligned nanometric fibers helped the cells in their orientation and growth, compared to the micrometric ones. Moreover, the production of collagen 1α1, decorin, tenomodulin and scleraxis was inhibited in the micrometric fibers [74,198]. 

Sahoo et al. evaluated bone marrow stem cell (BMSCs) proliferation, on random nanofibrous mats of PLGA, loaded with bFGF for tendon and ligament regeneration. The bioactive bFGF could activate tyrosine phosphorylation signaling within seeded BMSCs. The bFGF-releasing nanofibrous scaffolds facilitated BMSC proliferation, production and deposition of collagen and tenascin-C, and induced tendon/ligament-like fibroblastic differentiation [76]. Hayami et al. produced mats of aligned microfibers of PCLDLLA. They embedded the mats in a noncell-adherent photo-crosslinked N-methacrylated glycol chitosan hydrogel seeded with primary ligament fibroblasts. Ligament fibroblasts remained viable throughout the four-week culture period (72 ± 4%), and produced proteins such as collagen Types I and III, and decorin [115]. Surrao et al. produced aligned nanofibers of P(LLA-CL), PLDLA, PDLLA and PLLA for tendon and ligament applications. They compared the as-spun mats, with the crimped ones, obtained by immersing them in phosphate buffer saline (PBS). They investigated the effect of crimping both in terms of mechanical properties, and in terms of cell viability. They also found that the degree of crimping (amplitude and wavelength) was tunable by adjusting the difference between operating temperature and glass-transition temperature of the polymers. Crimping helped the nanofibers in recovery after the application of cyclic loads and increased the values of proliferation of the extracellular matrix production after 14 days and 8 weeks of culture with bovine fibroblasts [53,57]. 

**Figure 9:**
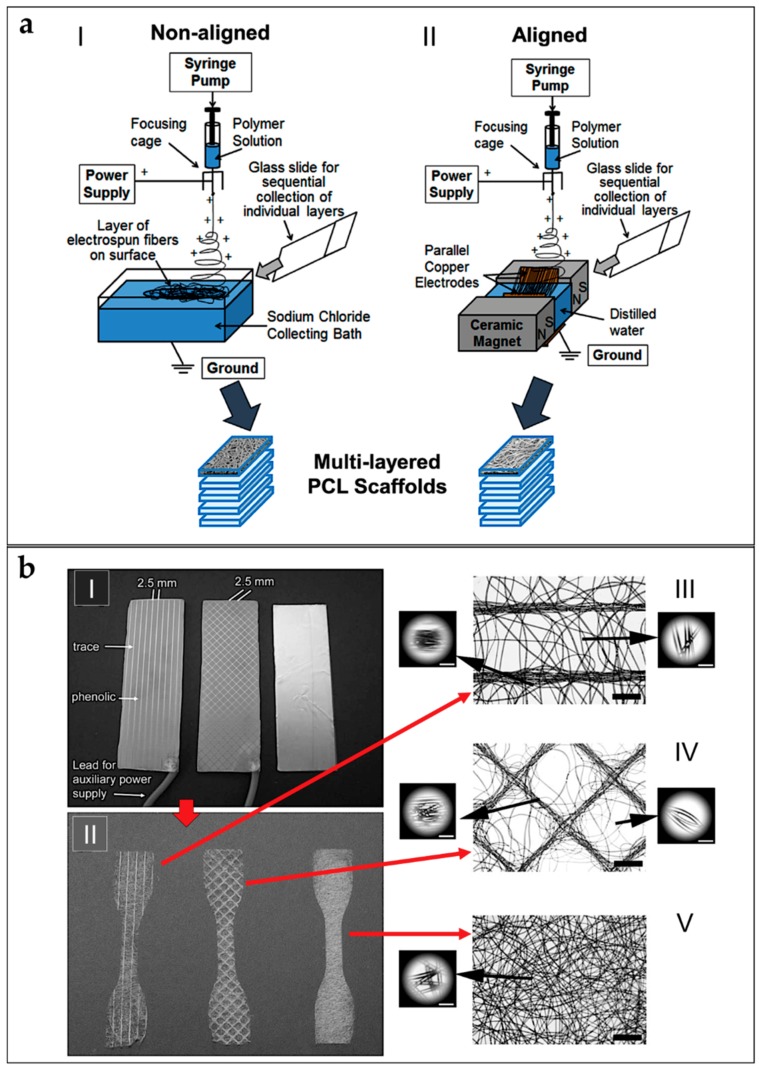
Different scaffolds produced as in vitro test bench for cell cultures. (**a**) Setup to produce PCL multi-layer scaffolds with liquid bath collectors for rotator cuff tissue engineering (adapted from Orr et al. [99], reproduced with permission. Copyright 2015, Elsevier): (I) setup to produce multilayer random nanofibrous scaffolds; and (II) setup to produce multilayer aligned nanofibrous scaffolds. (**b**) Nanofibrous mats of PU obtained by different pattern collectors (adapted from Karchin et al. [117], reproduced with permission. Copyright 2012, John Wiley and Sons): (I) mats of nanofibers are electrospun on a flat plate collector with different superficial patterns; (II) scaffolds prepared for tensile testing; and (III–V) SEM images of the different nanofibers scaffolds (scale bar: zoom out = 1 mm; zoom in = 100 micrometers).

Karchin et al. cultured with pig anterior cruciate ligament fibroblasts, different electrospun microfibrous mats of PU, obtained by electrospinning the nanofibers on flat plate collectors with different patterns (Figure 9a). The mechanical tests demonstrated tunable mechanical properties as a function of the templated architecture. Pig ligament fibroblast seeded scaffolds were subjected to periods of cyclic strains, of 1 h each, in a bioreactor. They found a statistically significant increment of collagen Type I gene expression, when stimulated at 3% strain at 0.5 Hz [117]. 

Tu et al. produced aligned PLLA and Coll core–shell nanofibers, aligned mats for tendon tissue regeneration. They used the PLLA as core polymer and the Coll as shell. The contact angle showed higher wettability of the core–shell nanofibers (47.93 ± 2.09°) compared to the native PLLA (104.52 ± 4.09°). Wide angle X-ray diffraction measurements confirmed the levels of crystalline orientation of 75.3% [62]. Chainani et al. electrospun PCL random microfibers mats on a saline bath, covered, respectively, by phosphate buffer saline (PBS), fibronectin or tendon-derived extracellular matrix. Scaffolds were maintained in culture with human adipose stem cells (hASCs). The collagen content was statistically greater by Day 28 in tendon-derived extracellular matrix scaffolds. The Young’s modulus did not statistically change over time, but yield strain increased with time in the cultures on the fibronectin and tendon-derived extracellular matrix mats. Histology demonstrated cell infiltration through the full thickness of all scaffolds [95].

Cardwell et al. produced random and aligned mats of fibers of PEUUR in different concentrations and diameters (the fiber diameters divided into: small <1 µm; medium = 1–2 µm; and large >2 µm) as a tendon and ligament tissue engineering applications, and studied the mouse fibroblast viability. Their found that the fiber diameter affects cellular behavior more significantly than fiber alignment. Initially, the cell density was greater on the mats of small fibers, but similar cell densities were found on all mats after an additional week in culture. After two weeks, gene expression of collagen 1α1 and decorin was increased on all mats. Expression of the tendon/ligament transcription factor scleraxis was suppressed on all electrospun mats, but expression on the large-diameter fiber mats was consistently greater than on the medium-diameter ones [119]. 

Full et al. applied a co-axial electrospinning to produce random and aligned nanofibers with a core of PU and a shell of a blend of PLGA (50:50 and 85:15) and Coll for ligament tissue regeneration. Different typologies of PLGA in blend with the Coll were investigated. They studied the mats mechanical properties and human foreskin fibroblasts (HFF) proliferation until 14 days of culture. They found higher mechanical properties for the mats of aligned nanofibers of PLGA (50:50)/Coll-PU compared to the PLGA (85:15)/Coll-PU ones. Moreover, they found a statistically significant increment of the cell adhesion in the aligned mats of PLGA (50:50)/Coll-PU compared to the other compositions and fiber organizations [79]. 

Sheikh et al. investigated random and aligned mats of nanofibers blends of Carbothane™ and different percentages of MWCNTs (0.06%, 0.33%, and 0.66%), for tendon and ligament grafts. The biocompatibility and cell attachment of the mats were investigated while culturing them in the presence of NIH 3T3 fibroblasts. The results indicated a non-toxic behavior and significant attachment of cells towards nanofibers for all the different compositions after seven days of culture. Promising mechanical properties were found for the aligned mats, especially for the nanofibers with 0.33% of MWCNTs that reached a failure stress of 72.78 ± 5.5 MPa [130]. 

Zhang et al., as application for tendon tissue engineering, compared the mechanical properties and the cell proliferation, in an in vivo rat Achilles’ tendon model, of random and aligned microfiber blends of PLLA, PEO and small molecule TSA. The in vivo implantation confirmed that TSA promoted the structural and mechanical properties of the regenerated Achilles tendon. However, the mechanical properties of the mats were weaker than the natural tendon [64].

#### 6.1.2. Multilayered and Co-Electrospun Scaffolds

Orr et al. used a modified liquid bath collector setup to obtain both random and aligned multilayer scaffolds of PCL microfibers for rotator cuff repair. For the random scaffolds, 70 layers of microfibers were overlapped. For the aligned scaffolds, 140 aligned layers were overlapped onto each other to obtain the final scaffold (Figure 9b). Then, scaffolds were cultured with hASCs for 0, 4, 7, 14 and 28 days. They evaluated that multilayered aligned scaffolds enhanced collagen alignment and tendon-related gene expression compared to multilayered nonaligned scaffolds. They also tested the mechanical properties of the scaffolds after 0 and 28 days of culture. Aligned scaffolds displayed increased expression of tenomodulin and exhibited aligned collagen fibrils throughout the full thickness, which increased yield stress and Young’s modulus of cell-seeded aligned scaffolds along the axis of fiber alignment [99]. Deepthi et al. obtained a scaffold by combining co-electrospinning and multilayer electrospinning, for ligament tissue regeneration. First, random and aligned nano/microfibrous mats were obtained, co-electrospinning PCL with different flow rates. The mats were then covered by a CTS/HA layer, and finally crosslinked. Better protein adsorption was found on the coated scaffolds, compared to the uncoated ones after 24 h of test. Moreover, the coated scaffolds improved the rabbit ligament fibroblast cell attachment and elongation along the aligned fibers after seven days of culture [91]. Yang et al. analyzed a multilayer electrospun scaffold, made by overlapping five co-electrospun microfibrous aligned mats of PCL and methacrylated gelatin, for tendon tissue regeneration. The scaffolds were then soaked with a photo-initiator and crosslinked by visible light. Photo-crosslinking was able to integrate stacked scaffold sheets to form multilayered constructs that mimic the structure of native tendon tissues. hASCs impregnated into the constructs remained responsive to topographical cues and exogenous tenogenic factors, such as TGF-b3. They also found statistically increased values of load to failure and Young’s modulus on the crosslinked ones [100]. Thayer et al. obtained a scaffold electrospinning aligned nanofibrous mats of PEUUR and PCL blends (0/100, 75/25 and 100/0) and then immersed in Coll gels, for ligament tissue engineering. The mechanical testing of the mats showed Young’s moduli of 15 ± 4.0 MPa (100/0 PEUUR/PCL), 31 ± 14 MPa (0/100 PEUUR/PCL) and 5.6 ± 2.5 MPa (75/25 PEUUR/PCL). Rat bone marrow mesenchymal stem cells (BMMSCs), seeded for 14 days in the collagen hydrogel phase, were oriented by the network. Systematic variation of fiber modulus affected expression of α-smooth muscle actin and Scleraxis [80]. Gurlek et al. electrospun PCL nanofiber random mats for anterior cruciate ligament regeneration, and studied their mechanical properties, which resulted lower than the ones of anterior cruciate ligament [89]. Dodel et al. produced a scaffold made of three different layers for ligament regeneration. First, a mat of aligned SE electrospun nanofibers was placed on a flat plate collector, and used to electrospun a random PEDOT nanofiber mat. Finally, the bilayer nanofibrous scaffold was embedded in a CTS sponge. Unrestricted somatic human stem cells (USSCs) were cultured. The effect of DC electric pulses to cells cultured on polymer was assessed. Cellular function was more active in scaffolds with electrical induction, where collagen Type I, collagen Type III, decorin, biglycan and aggrecan genes were intensively expressed [123]. 

### 6.2. Patches and Augmentation Grafts

Another way to try to increase the mechanical properties of the electrospun scaffolds, for tendon and ligament tissue engineering applications, is to apply different techniques derived from textiles. In some works, researchers have produced composite scaffolds, combining non-electrospun knitted structures, and electrospun nanofiber layers. 

#### 6.2.1. Patches

**Figure 10:**
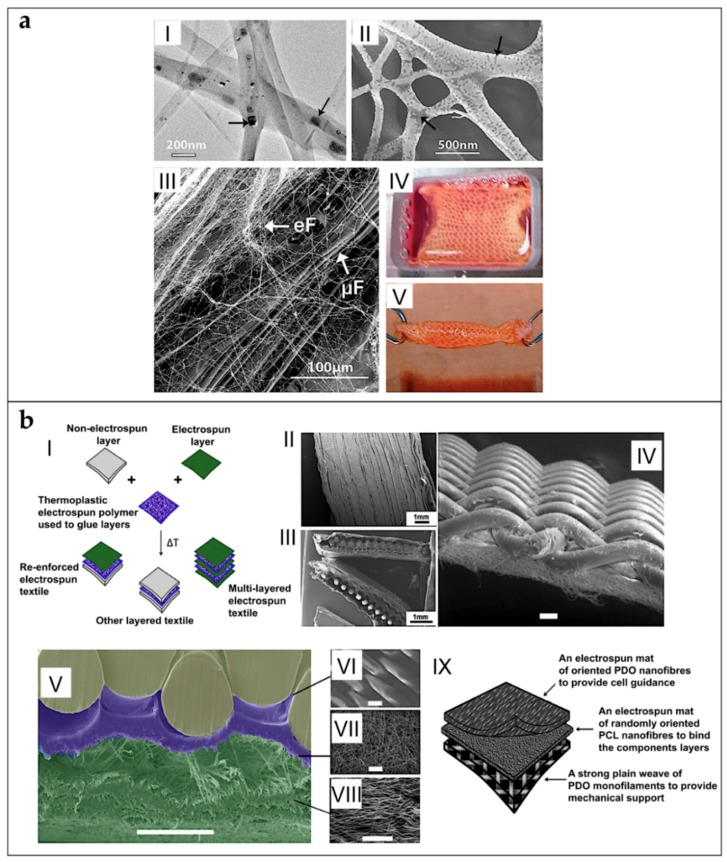
Different scaffolds used as *in vivo* patches or augmentations for tendon and ligament tissue engineering. (**a**) Microfiber SE knitted scaffolds, mounted on a drum collector, and covered by nanofibers of PLLGA loaded with bFGF (adapted from Sahoo et al. [84], reproduced with permission. Copyright 2010, Elsevier): (I–II) SEM images of nanofibers at different magnifications (arrows point bFGFs particles); (III) SEM image of nanofibers (eF) and SE microfibers (μF); and (IV–V) images of the complete scaffold before (IV) and after (V) twisting. (**b**) Multilayer scaffold for tendon repair, made of a woven layer of PCL monofilament and electrospun nanofibers mats of random nanofibers of PCL and aligned PDO nanofibers, obtained using a drum collector at different rotation speeds (adapted from Hakimi et al. [106], reproduced with permission. Copyright 2015, Elsevier): (I) schematic assembly procedure; (II–VIII) SEM images of the scaffold and mats ((IV–VIII) scale bar = 100 micrometers); and (IX) explanation of the different mats functions.

Sahoo et al. developed a scaffold for tendon/ligament tissue engineering applications, by covering with random electrospun nanofibers of PLGA, a knitted scaffold, composed by microfibers yarns of PLGA. Porcine bone marrow stromal cells were seeded onto the scaffolds. Cell proliferation was faster in these scaffolds compared to the only knitted ones. Moreover, cellular function was more active, with significant expression of the collagen Type I, decorin, and biglycan genes. However, the failure load of the nanofibrous coated scaffolds after ageing in PBS (7 days = 18.11 ± 3.53 N; 14 days = 2.26 ± 0.57 N) was lower than the tendons and ligaments [77]. To reproduce a tendon or a ligament, Sahoo et al. covered a knitted SE scaffold, mounted on a rotating drum collector, with random nanofibers of PLLGA loaded with bFGF. After this process, the scaffolds were twisted in yarns, and seeded with bone marrow stromal cells (BMStCs) (Figure 10a). The nanofibers coating sustained release of bFGF, initially stimulating mesenchymal progenitor cell (MPCs) proliferation, and, subsequently, their tenogenic differentiation. Up-regulated also gene expression of ligament/tendon-specific extracellular matrix proteins and increased collagen production. This contributed to enhancing the average failure load (83 N) of the construct after three weeks of cellular culture, reaching values similar to the rabbit medial collateral ligament (88 N). However, the average stiffness of the scaffolds (6.97 MPa) after cellular culture was lower than the natural ligaments (46–47 MPa) [84]. Vaquette et al. electrospun a microfibrous aligned mat of P(LLA-CL), on two different knitted structures of PLLGA and SE. The knitted scaffolds were fixed longitudinally on the surface of a high-speed rotating drum collector, and covered by a layer of nanofibers. The mechanical tests exhibited an initial toe region and a Young’s modulus similar to the ones of human ligaments. Rat BMMSCs proliferated on the composite scaffolds and orientated along the direction of microfibers alignment. Cells produced collagen Types I and III [54].

Beason et al. focused on rotator cuff repair: they produced mats of aligned nanofibers co-electrospinning PCL and PEO (as sacrificial fibers). A rat rotator cuff model was used to investigate the in vivo performance. The scaffolds remained in place, with more noticeable cellular infiltration and colonization lacking sacrificial fibers. Biomechanical testing revealed reduced mechanical properties in relation to the increased cross-sectional area, caused by the extra thickness of the implanted scaffold material [96]. 

#### 6.2.2. Augmentation Grafts

Sharifi-Aghdam et al. focused on tendon tissue engineering: they electrospun a random nanofibrous blend of PU/Coll on a knitted SE scaffold of non electrospun yarns. The tensile tests on samples including blend nanofiber and knitted SE indicated that PU/Coll-coated knitted SE had appropriate mechanical properties in terms of Young’s modulus (525 ± 23 N). The Alamar Blue assay on the L929 fibroblast cell line demonstrated appropriate cell viability, with a significant proliferation on the scaffold containing more Coll content [118]. 

Ni et al. electrospun nanofibers of SE annealed in methanol for Achilles tendon augmentation. They compared, in an in vivo rabbit model, the different effects of healing of the SE patches, wrapped on the injured Achilles’ tendons, and fixed with different suture techniques. After 28 days, they found that a surgical scenario, where the standard suture was augmented by the photobonded silk wrap, may provide optimal mechanical strength [128]. Inui et al. used an in vivo rabbit rotator cuff model, to test the behavior of PDLLGA random microfibers. They tested the mechanical and biological performances of the grafts at different time points until 16 weeks, finding progressively increment of the patch mechanical properties (failure load at: Week 0 = 5.4 ± 2.5 N; Week 16 = 75.3 ± 18.7 N), tissue regenerations and cells migration inside the patch [75]. Manning et al. tested in an in vivo canine model for tendon repair, a multi-layered scaffold made of aligned nanofibrous mats of PCL in combination with fibrin/heparin-fibrin-based delivery system layers (filled also with mesenchymal stem cells). The in vitro study showed that the cells remained viable and that a sustained growth factor release was achieved. The in vivo study confirmed that cells remained viable in the tendon repair environment after nine days after implantation. No negative reaction was seen at dissection or based on the mRNA level. However, a mild immune response was detected histologically [98]. Zhao et al. developed random nanofibrous membranes of PLGA loaded with bFGF for rotator cuff repair. They tested the mats in an in vivo rat rotator cuff model. After surgery, the electrospun membranes increased the area of glycosaminoglycan staining at the tendon–bone interface compared with the control group, and bFGF–PLGA improved collagen organization. Biomechanical testing showed that the electrospun membrane had a greater ultimate load-to-failure and stiffness than the control group at four and eight weeks [81]. Zhao et al. embedded random nanofibers mats of PLLA in GT, and tested the scaffolds in an in vivo rat rotator cuff repair model. Histologic observations revealed that GT-PLLA membranes have proper biocompatibility and biodegradability. At eight weeks postoperatively, the area of glycosaminoglycan staining at the tendon–bone interface was increased compared to the control group and improved collagen organization. Biomechanical testing revealed that the GT-PLLA group had a greater average failure load (>30 N) and stiffness (>12.5 MPa) than the control group (failure load <30 N; stiffness about 12.5 MPa) [65]. 

To obtain random nanofibers patches for rotator cuff repair, Zhao et al. co-electrospun PCL and CTS, and testing them in vivo in a rat model. The composite scaffolds had improved strength and failure strain compared to the control CTS scaffolds and increased stiffness compared to the control PCL scaffolds. Moreover, they demonstrated better fibroblast attachment and proliferation compared to the PCL scaffolds. Radiological and histological analysis revealed that the PCL-CTS scaffolds promoted new bone formation and collagen and glycosaminoglycan expression compared to the control [97]. Zhang et al. developed, for the regeneration for the Achilles tendon, random and aligned mats of nanofibers of a solution of CTS, GT, PLLA and PEO. The scaffolds were seeded with human-induced pluripotent stem cells (hiPSCs). The in vivo rat tendon repair study confirmed that aligned fiber scaffold with hiPSCs improved the structural and mechanical properties of tendon injury repair [63]. Hakimi et al. developed a three-layers scaffold for tendon repair. The scaffold consisted of a woven layer of PCL monofilament, overlapped on a mat of random nanofibers of PCL, and an aligned mat of PDO nanofibers (Figure 10b). The mechanical properties were in the same range as the human rotator cuff. The in vivo rotator cuff rat model showed that all animals developed fibrous capsules around the repaired tendons. At 12 weeks post-operation, the fibrous tissue appeared more compact and tightly adhered to the material [106].

### 6.3. Multiscale Hierarchical Scaffolds for Massive Replacement

Tendons and ligaments have a three-dimensional morphology and different shapes depending on the anatomical site. For this reason, some researchers have tried to develop scaffolds able to reproduce the entire hierarchical morphology of tendon and ligament tissue.

#### 6.3.1. Fascicle-Inspired Bundles and Yarns

A common approach is to use electrospun bundles and yarns as a basic “brick” to mimic the fascicles of tendons or ligaments [5,6,7,9]. 

**Figure 11:**
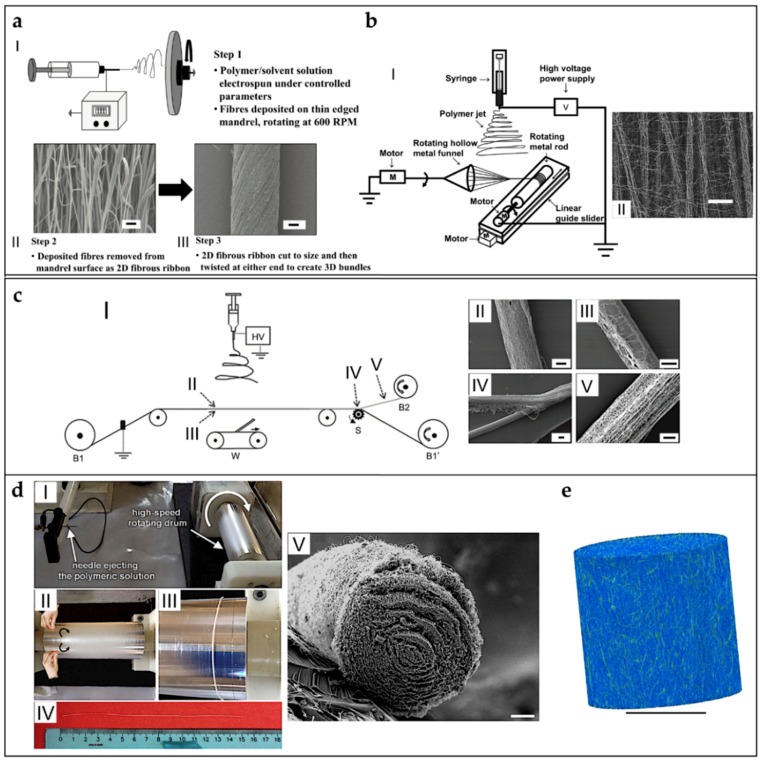
Different setups to produce nanofibrous bundles and yarns. (**a**) Yarns of PCL and PLGA, for tendon tissue regeneration (adapted from Bosworth et al. [82], reproduced with permission under the terms of the CC BY 3.0 license. Copyright 2014, Hindawi): (I) mats of nanofibers are electrospun on a high-speed rotating drum collector and then ribbons of the mat are cut and twisted to obtain the yarns; and (II–III) SEM images of the nanofibers and of a yarn (scale bar II = 2 micrometers; scale bar III = 50 micrometers). (**b**) Electrospinning setup to produce mats composed by micro-yarns of a blend of P(LLA-CL) and SF, for tendon tissue engineering (adapted from Yang et al. [56], reproduced with permission. Copyright 2014, Elsevier): (I) nanofibers are collected between the funnel collector and the rotating drum collector and the combination of the two rotating collectors allows producing a mat of micro-yarns and random nanofibers; and (II) SEM image of the micro-yarns (scale bar = 100 micrometers). (**c**) Automatic electrospinning setup to produce continuous bundles of PDO (adapted from Mouthuy et al. [124], reproduced with permission under the terms of the CC BY 3.0 license. Copyright 2014, IOP Publishing): (I) electrospinning machine, in which a metal rod is covered by nanofibers, and, in the final part, the electrospun mat is separated from the rod; and (II–IV) SEM images of a bundle during different points of the process (scale bars = 100 micrometers). (**d**) Process to produce finite length bundles of nanofibers of PLLA, PLLA/Coll blends (adapted from Sensini et al. [59], reproduced with permission. Copyright 2017, IOP Publishing): (I) aligned nanofibers are collected on a high-speed rotating drum; (II–III) mats are manually wrapped on the drum to obtain bundles; (IV) the bundles are removed from the drum; and (V) SEM image of a section of a bundle (scale bar = 50 micrometers). (**e**) High resolution X-ray tomographic image at 0.4 micrometers voxel size of a PLLA bundle (adapted from Sensini et al. [60], reproduced with permission. Copyright 2018, John Wiley and Sons) (scale bar = 200 micrometers).

Xu et al. produced aligned nanoyarns mats of P(LLA-CL) and Coll blends, for tendon tissue applications [173]. In this work, the nanofibers, twisted in nanoyarns by the vortex (adapted from Teo et al.), were collected on a rotating drum producing a mat of nanoyarns. Finally, the mats were cultured with rabbit tendon tenocytes, that showed increased values of proliferation after 14 days in static culture [55]. Bosworth et al. produced yarns of nanofibers of different polymers, such as PCL and PLGA for tendon tissue regeneration. The nanofibers were aligned on a rotating drum collector, producing mats which were cut in ribbons, and manually twisted to obtain the yarns (Figure 11a). The yarns showed mechanical properties in the range of tendon fascicles. The in vitro cell culture both with equine fibroblasts (static), and human stem cells (dynamic), showed increased values of proliferation and collagen production. After 21 days of dynamic cell culture, the bundles increased their mechanical properties (failure stress about 50 MPa; Young’s modulus about 100 MPa) compared to the static culture at the same time point (failure stress about 20 MPa; Young’s modulus 80–90 MPa) [82,101,102]. 

Thayer et al. focused on ligament regeneration: they electrospun blends of PLGA and PEGDA or PEUUR and PEGDA, obtaining random nanofiber mats. The mats were then rolled up on a mandrel, and crosslinked with PEG hydrogel network. A single mesh was rolled and injected of PEGDA and phosphate buffer saline (PBS) solution loaded with a photoinitiator. The scaffold was finally crosslinked with ultraviolet light to form acellular composites. They tested the cell viability with mouse mesenchymal stem cells, finding decreased vitality after five days of culture [78]. Yang et al. adapted the setup proposed by Lotus et al. to produce mats of aligned micro-yarns of a blend of P(LLA-CL) and SF [164]. After its formation, the yarn was continuously wrapped on the drum and covered by the same random nanofibers, producing the final mat (Figure 11b). The mats of micro-yarns were then seeded with BMMSCs, finding increased values of viability after 28 days of culture, compared with the control mat of aligned and random nanofibers. However the mechanical properties of the micro-yarns (failure stress = 24.25 ± 0.76 MPa; Young’s modulus = 288.95 ± 13.26 MPa) resulted lower compared to the aligned nanofibers (failure stress = 39.10 ± 2.89 MPa; Young’s modulus = 433.6 ± 48.1 MPa) [56]. 

Mouthuy et al. developed an automated system, to produce continuous nanofiber bundles of PDO, electrospun on a metallic wire for tendon tissue engineering applications. After the coating with aligned nanofibers, a cutter wheel divided the wire collector from the bundle that was collected on a rotating drum (Figure 11c). The continuous bundle was cut in pieces that were twisted together. The average failure load of the single bundle was about 1 N. After eight days of culture in vitro with human tenocytes, the bundle showed cell attachment [124].

Cook et al. tested in a bioreactor hierarchical scaffolds for tendon and ligament applications: bundles of microfibers of PEO/fibrinogen loaded with adipose derived stem/stromal cells were investigated. The bundles, with different degrees of porosity, were obtained by electrospinning the solution in a liquid bath vortex collector containing CaCl_2_, glucose and thrombin. They demonstrated that cells proliferation after 21 days was as higher as the bundle porosity was increased. They also monitored the strain distributions with fluorescent digital image correlation [121]. 

Domingues et al. produced nanofibrous bundles of a PCL/CTS blend, for tendon tissue regeneration, loaded with CNCs. They found that small CNC contents in the bundles improved the limited tensile properties (strength and stiffness) while preserving their ductility [104]. Levitt et al. addressed tendon applications: they studied the changes in mechanical properties of nanofibrous yarns obtained by a funnel collector derived from Ali et al. [178]. PCL, PAN and PVDF-TrFe yarns were produced at different funnel rotational speed and mechanically characterized, finding that PVDF-TrFe and PCL yarns have a higher failure strain than PAN yarns [105]. Pauly et al. produced bundles of aligned and random nanofibers of PCL for ligament tissue engineering, by wrapping on a drum collector sections of the mats obtained. They also characterized the mechanical properties of the bundles, finding values close to human anterior cruciate ligament fascicles. Finally, they tested the cell proliferation with hASCs, finding increased values of proliferation after seven days of culture [92].

Sensini et al. developed a versatile method of production of bundles for tendon and ligament tissue regeneration and replacement. They characterized the morphology, the mechanical properties and the cells growing, of electrospun nanofibrous bundles of PLLA, PLLA/Coll blends and Nylon 6.6 (just morphologically characterized), obtained by wrapping the aligned mats on a drum collector (Figure 11d). They found that the resorbable bundles (PLLA and PLLA/Coll blends) had mechanical properties close to the human tendon fascicles. The internal morphology of the bundles and the directionality of the nanofibers (evaluated with high resolution X-ray tomography) was similar to the human fascicles one (Figure 11e). In addition, the cell viability tests with human tenocytes showed increased levels of proliferation on the PLLA/Coll blends after 14 days of culture [59,60].

Bhaskar et al. tested in an in vivo mouse model, the yarns previously developed by Bosworth et al. for tendon tissue regeneration [82,101,102]. To evaluate the effects of different sterilization approaches, different groups of scaffolds were sterilized using gamma irradiation and ethanol immersion before the implantation. Cell infiltration and proliferation were performed to determine the effect on cell response over a six-week period. Immunohistochemical analysis was performed to characterize inflammatory response, cell proliferation, collagen deposition, myofibroblast activity, and apoptosis. Both sterilization techniques did not significantly affect the cell response [103].

#### 6.3.2. Hierarchically Structured Scaffolds

To increase the hierarchical organization and the mechanical properties of their scaffolds, some researchers tried to match multiple mats, bundles or yarns together by twisting or braiding them. Barber et al. focused on tendon and ligament tissue engineering: they braided a different number of electrospun bundles (3–5) of PLLA and tested their mechanical properties and cell viability with human mesenchymal stem cells (hMSCs) in a bioreactor. They tested the mechanical properties to measure the Young’s modulus (three bundles = 55.0 ± 2.8 MPa; four bundles = 47.8 ± 7.5 MPa; five bundles = 47.6 ± 2.8 MPa) and failure stress (three bundles = 7.62 ± 0.2 MPa; four bundles = 6.57 ± 0.5 MPa; five bundles = 6.67 ± 0.4 MPa). They also found an up-regulation of the production of collagen Types I and III by the cells, during the cyclic stimulation in bioreactor [61]. Bosworth et al. used the yarns previously described [82,101,102], and produced a braided scaffolds for tendon regeneration [82]. Rothrauff et al. studied scaffolds made by dog-bone mats of aligned nanofibers of PLLA or PCL, for tendon and ligament tissue engineering. The mats were obtained electrospinning on a rotating drum collector, and finally braiding them together. They compared the mechanical properties and the BMMSCs infiltration of the braided structures, with multilayered scaffolds of the same materials. The failure load for the braided PCL scaffolds was 164.82 ± 11.13 N and for the stacked ones was 94.67 ± 6.7 MPa. The Young’s modulus for the braided PCL scaffolds was 45.96 ± 10.03 MPa and for the stacked ones was 66.48 ± 11.29 MPa. The failure load of the braided PLLA scaffolds was 27.51 ± 4.40 MPa and for the stacked ones was 30.03 ± 1.57 MPa. The Young’s modulus for braided PLLA was 45.57 ± 38.96 MPa and for the stacked ones was 118.47 ± 21.81 MPa. Cell proliferation was higher in the stacked scaffolds [58]. In another study on ligament tissue regeneration, Pauly et al. grouped in parallel bunches and conjugated with CTGF the bundles previously described [92]. They studied the ovine bone-marrow derived mesenchymal stem cells (OBMSCs) proliferation. They found immediately increased values cell viability and collagen expression compared with the unconjugated control [93].

**Figure 12:**
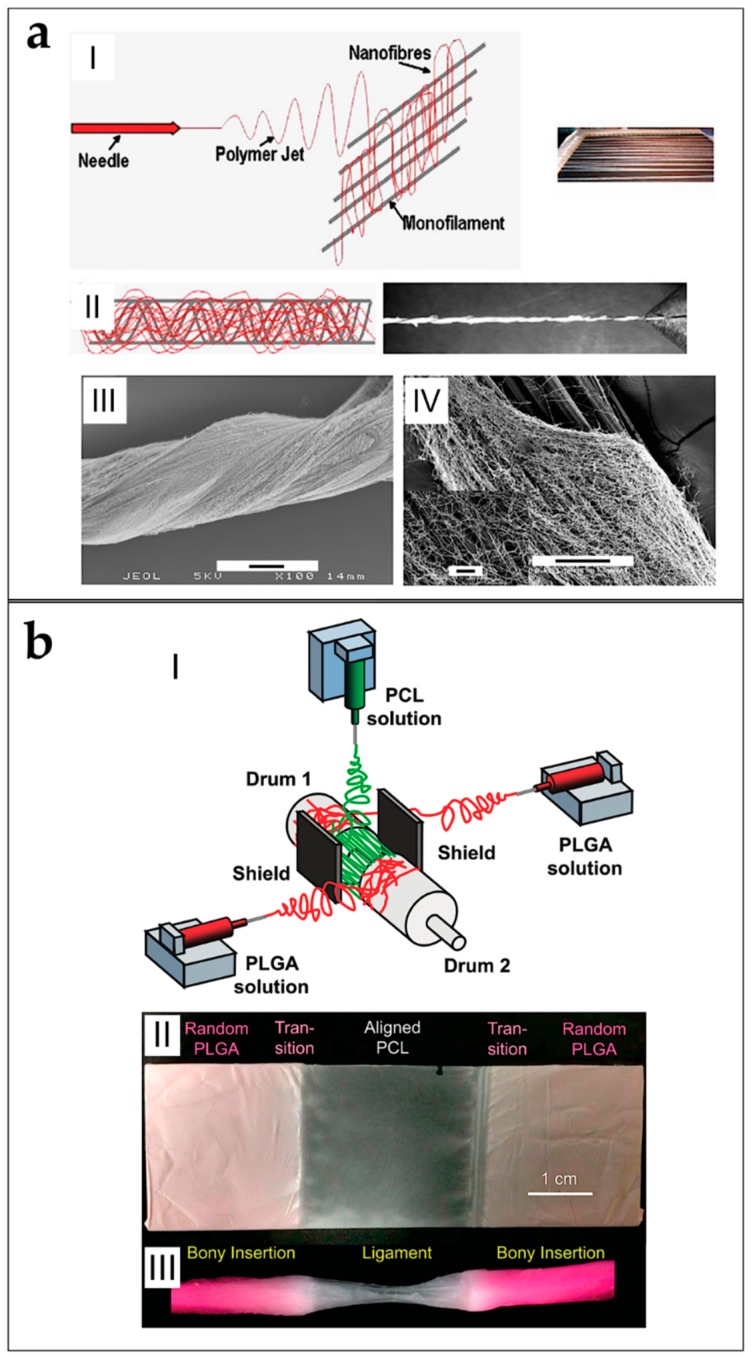
Schematic workflow to produce multiscale hierarchically structured scaffolds for tendon and ligament tissue engineering. (**a**) Microfilaments of PA covered by electrospun nanofibers of PEO, and manually twisted to obtain a scaffold for tendon and ligament applications (adapted from Zhou et al. [122], reproduced with permission. Copyright 2010, Elsevier): (I) the microfilaments are fixed parallel on a rectangular structure, and over pose to a flat plate collector. Then, the nanofibers are electrospun on the microfilaments; (II) after the electrospinning session, the microfilaments are twisted to obtain a tendon/ligament like structure; and (III–IV) SEM images of the scaffold (scale bar III = 100 micrometers; scale bar IV = 100 micrometers, zoom-in = 10 micrometers). (**b**) Combination between air gap collector setup and co-electrospinning of PLLGA and PCL to produce a random/aligned nanofibrous scaffold for ligament tissue regeneration (adapted from Samavedi et al. [87], reproduced with permission. Copyright 2014, John Wiley and Sons): (I) schematic representation of the electrospinning setup; (II) image of the random–aligned–random nanofibrous mat after the removal from the collector; and (III) final scaffold after the wrap of the mat of nanofibers.

Other research groups developed scaffolds able to reproduce the hierarchical structure of tendons and ligaments by grouping the structures inside the scaffolds with electrospun sheaths, trying to mimic the epitenon or epiligament tissue. Zhou et al. morphologically characterized a scaffold, suitable as artificial tendon/ligament. First, a nanofibrous random sheath of PEO, was electrospun on a cardboard frame, in which were placed a parallel group of poly(amide) PA monofilaments. After the coverage, the monofilaments were twisted together to obtain a structure similar to a tendon or ligament. They also braided some of these structures to increase the hierarchical assembly (Figure 12a). However, they just briefly characterized the scaffold in terms of abrasion tests [122]. To mimic the tendon or ligament morphology, Naghashzargar et al. produced a random nanofibrous sheath of PCL or P3HB, on aligned SF yarns, fixed in a wooden structure, above the flat plate collector. After the deposition the SF yarns were twisted to obtain the final scaffold. They found that the nanofibrous sheath slightly increased the failure load of the scaffolds compared to the uncoated SF yarns (SF = 92.6 ± 8.2 N; SF-P3HB = 97.6 ± 11.4 N; SF-PCL = 110.5 ± 6.6 N). No cytotoxic effects on the murine fibroblast cells were found [88]. 

Some works aimed to replicate the hierarchical structure of the whole tendon, and the bone–ligament complex, electrospinning them with the gap collector technique. Samavedi et al. designed a nanofibrous scaffold that tried to morphologically reproduce a complete bone-ligament-bone complex (Figure 12b). Aligned PCL nanofibers were used to mimic the ligament tissue, instead PLLGA random nanofibers were used to reproduce the bone tissue. The scaffold was produced by alternating the electrospinning of the solutions on two drum collectors and on a gap between them. They tested the cell proliferation with BMStCs on the planar mats with gradients of random and aligned nanofibers, finding random orientations on the random PLGA regions, and high alignment on the aligned PCL regions. Finally, they tested the mechanical properties of both the mats and the three-dimensional scaffolds. They found stress concentrations in the aligned PCL region of the three-dimensional scaffolds, and lower mechanical properties compared to the flat mats [87].

**Figure 13:**
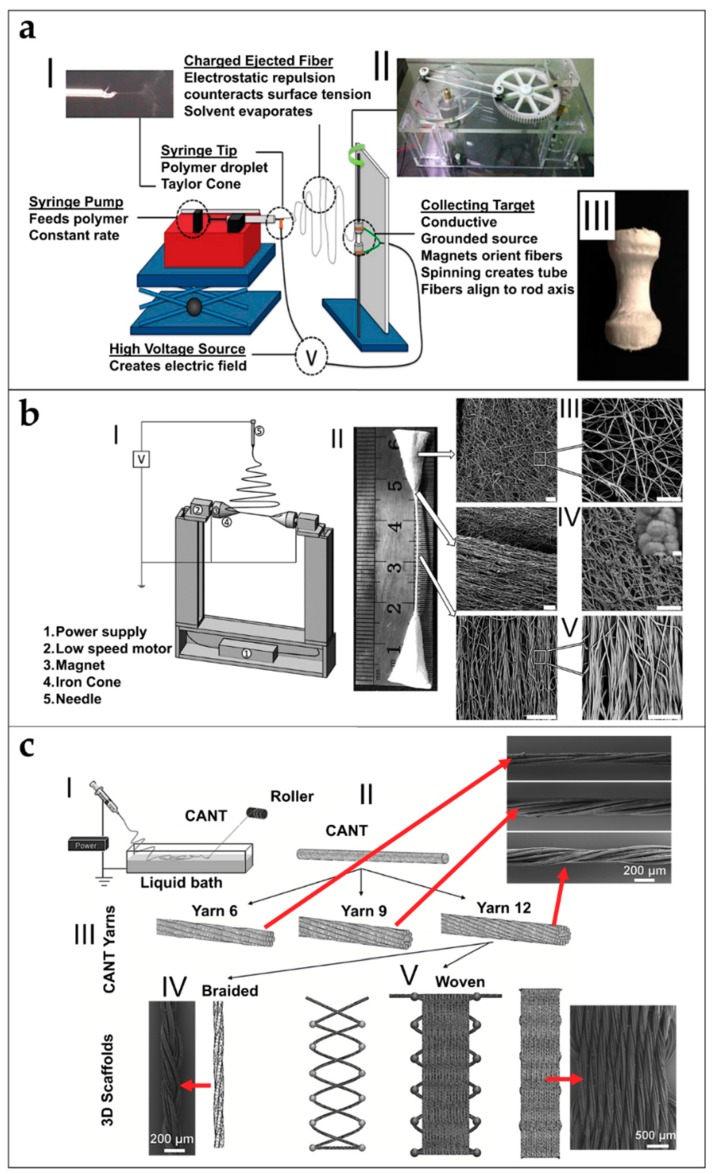
Schematic workflow to produce multiscale hierarchically structured scaffolds for tendon and ligament tissue engineering: (**a**) Modified gap collector setup to produce a scaffold for tendon tissue regeneration (adapted from Banik et al. [107], reproduced with permission. Copyright 2016, Springer Nature): (I) schematic representation of the electrospinning setup; (II) image of the motorized setup of the collector; and (III) image of the final scaffold. (**b**) Bone–ligament–bone nanofibrous scaffold of PCL, obtained by a modified air gap collector (adapted from Lin et al. [114], reproduced by permission of The Royal Society of Chemistry. Copyright 2017); (I) picture of the electrospinning setup; (II) image of the final scaffold; (III–V) SEM images of the nanofibers in the different parts of the scaffold ((III–IV) scale bar = 10 micrometers; (V) scale bar = 10 micrometers (right image) and 5 micrometers (left image)). (**c**) Two different multiscale scaffolds made of braded or woven bundles blend of PCL/CTS, loaded with CNCs for tendon tissue engineering (adapted from Laranjeira et al. [108], reproduced with permission. Copyright 2017, John Wiley and Sons); (I–II) schematic picture of the process to obtain the continuous bundles by a liquid bath collector; (III) 6, 9 and 12 bundles are twisted together to obtain hierarchically structured yarns; (IV) SEM image and a schematic picture of the nanofibers hierarchically structured yarns braided together; and (V) SEM image and schematic picture of the woven multiscale scaffold.

Banik et al. produced a scaffold to simulate the whole tendon. A gap collector was made by two cylindrical rods able to rotate synchronously. To enhance the deposition and alignment of the PCL nanofibers, magnets were placed near the gap. The final scaffold was a cylinder of aligned or random nanofibers, depending to the rotational speed of the two cylindrical collectors (Figure 13a). They found lower properties compared to the tendon tissue (average Young’s modulus = 35.8 MPa; failure stress < 12 MPa). They also seeded hMSCs on the scaffolds, finding increased values of proliferation and no cytotoxic effects [107]. Lin et al. tried to reproduce the bone–ligament–bone complex, electrospinning PCL nanofibers on a motorized air gap conic collector setup (Figure 13b). As a result, they produced two random conic nanofibers mats on their surfaces, and an aligned nanofibrous region between the two tips of the cones. The conic nanofibrous ends were then mineralized with BLM. They tested the mechanical properties of the ligament-like bundle, finding values in the same range of the human ligament fascicles (failure stress = 38.7 ± 6.2 MPa; Young’s modulus = 82.8 ± 11.6 MPa). They also evaluated the cell proliferation of human BMMSCs, finding up-regulation of the tendon or bone markers in the aligned and random sites [114]. Recently, Laranjeira et al. produced nanofibrous tendons starting from electrospun bundles of a blend of PCL/CTS, loaded with CNCs thanks to a liquid bath collector. After that, groups of bundles were twisted together and finally braided or woven to obtain the final scaffolds (Figure 13c). They tested the mechanical properties of the scaffolds finding increased values of the yield stress of the braided scaffolds (42 ± 8 MPa) compared to the woven ones (33 ± 2 MPa). They also found increased collagen Types I and III after 7 and 21 days of culture with human tendon derived cells (hTDCs) and hASCs [108].

Mouthuy et al. manually twisted together the bundles previously described [124], and produced twisted structures for tendon replacement applications. They applied an annealing process at 65 °C to the twisted structures. The failure load of the twisted structure (of the order 20 N) was increased after the annealing process (average value > 20 N). They evaluated in vitro the changes in the mechanical properties after permanence in PBS, and found that, after 12 weeks, they were drastically reduced. They also found increased cell adhesion in the static culture compared with the single bundles. Finally they evaluated the performance of the twisted structure in an in vivo rat rotator cuff model, confirming the biocompatibility of the scaffold [124]. Vaquette et al. electrospun random nanofibrous mats of PCL for ligament tissue engineering applications. Three mats for each scaffold were wrapped and finally braided together. The mats were seeded with BMMSCs for four weeks and finally subcutaneously implanted in an in vivo rat model to evaluate the biocompatibility. While the biocompatibility and cell proliferation were promising, the mechanical properties after four weeks of static culture were significantly lower than the as-spun controls (average control failure load = 0.7 N; average four weeks scaffold failure load = 0.3 N) [94]. 

### 6.4. Bone Insertions

One of the critical points for clinical deployment is to provide adequate connection to the host bones (both for tendons and ligaments) or to the host muscle (tendons only). The insertion must provide enough strength, avoid stress concentrations, and promote tissue integration and growth. Some groups focalized their work on the mimicking and replacement of both the bone and the muscle insertion of tendons and ligaments.

**Figure 14:**
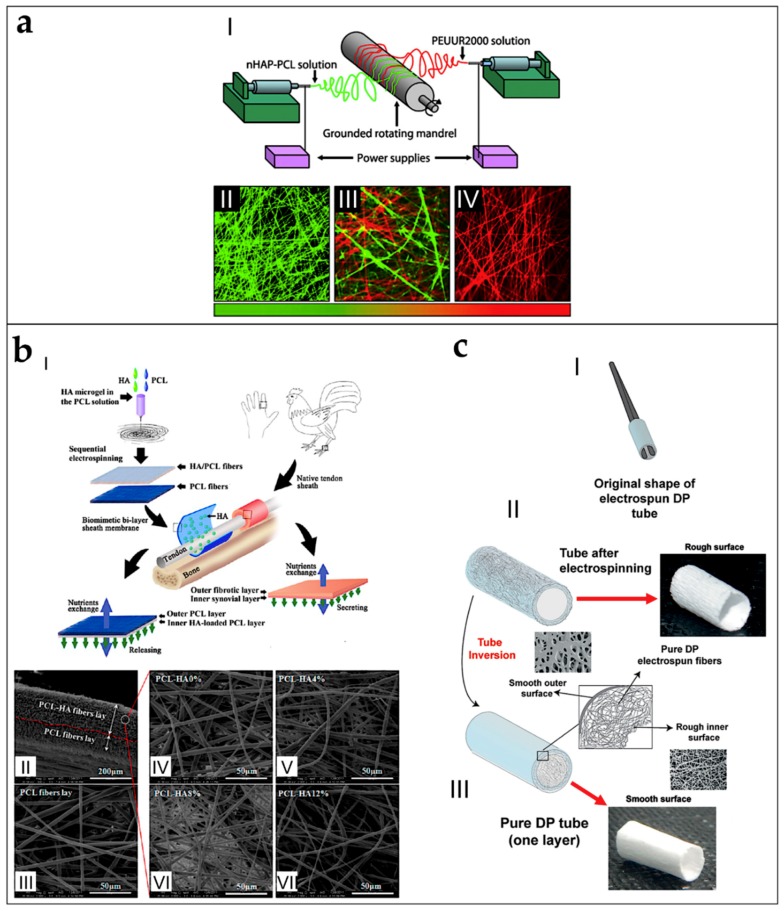
Schematic workflows to produce electrospun scaffolds suitable for tendon/ligament-to-bone attachment or for tendon and ligament healing and anti-adhesion applications. (**a**) Co-electrospun random mats with gradient of PEUUR2000 and PCL, loaded with nanohydroxyapatite (HAp) for ligament-to-bone regeneration (adapted from Samavedi et al. [110], reproduced with permission. Copyright 2011, Elsevier): (I) co-electrospinning setup to obtain nanofibrous mats with gradient on a rotating drum collector; (II) fluorescent image of the HAp-PCL nanofibers side; (III) fluorescent image of the transition region of the two nanofiber sides; and (IV) fluorescent image of the PEUUR2000 nanofibers side. (**b**) Double layer electrospun mat of random PCL and a blend of PCL/HA for tendon healing (adapted with permission from Liu et al. [113]. Copyright 2012, American Chemical Society): (I) electrospinning setup and in-situ application of the scaffold; (II) SEM section of the bilayer scaffold; (III) PCL nanofibers; and (IV–VII) layers of HA/PCL nanofibers with different percentages of HA. (**c**) Production process to obtain a nanofibrous tube or conduit of DP for tendon healing and anti-adhesion applications (adapted from Evrova et al. [127], reproduced with permission. Copyright 2016, John Wiley and Sons); (I) after the nanofibers are electrospun on a rotating drum collector, the tube is removed from it; (II) structure and images of the tube immediately after the movement from the drum collector; and (III) structure and images of the tube after is inversion.

Li et al. focused on the tendon-to-bone interface: they produced mats of PLGA and PCL nanofibers, mineralized with gradients of tricalcium phosphate. They found increased values of strain and decreased value of Young’s modulus due to the reduction of the mineralization. They seeded the scaffolds with mouse preosteoblast cells (MPC3T3), finding increased values of proliferation in the sites with presence of tricalcium phosphate [131]. Samavedi et al., to simulate the bone-to-ligament interface, studied a random nanofibrous scaffold with a gradient of PEUUR2000 and PCL, loaded with nanohydroxyapatite (HAp). The two solutions were co-electrospun on the side of the drum with a central overlap zone (Figure 14a). The progressive mineralization of the mat induced a change of the mechanical properties in terms of Young’s modulus (HAp-PCL = 2.4 MPa; PEUUR2000 = 0.23 MPa; mixed region = 0.55 MPa) and failure stress (HAp-PCL = 0.5 MPa; PEUUR2000 = 0.6 MPa; mixed region = 0.4 MPa). They obtained encouraging results in terms of MC3T3-31 osteoprogenitor cell metabolic activity [110]. In another work, Samavedi et al. applied the same principle to produce random nanofibrous mats with a gradient of PCL, loaded with nanohydroxyapatite (HAp), and PEUUR. They cultured the mats with BMSCs, and found that the presence of mineral in the electrospun scaffolds promoted the elevation of morphogenic protein-2 and protein-like osteopontin mRNA, while suppressing the expression of alkaline phosphatase mRNA. Immunofluorescent staining confirmed the presence of proteins like osteopontin and bone sialoprotein, indicating osteoblastic maturation by day 28. Moreover the mineral gradients could promote a spatial gradient of osteoblastic phenotype in BMSCs [111]. Xie et al. focused on the tendon-to-bone insertion: they developed a machine able to modulate the gradient of the random electrospun nanofibers produced. They started aligning nanofibers of PCL, thanks to a gap collector setup. Subsequently, they collected the aligned fibers on a glass coverslip, and put the mat in the machine to produce the gradient. The flat plate collector of the machine was motorized and permitted to moving the scaffold to cover just sections of the mat with random nanofibers still of PCL. Finally they cultured adipose derived stem cells (ADSCs) finding a differentiation of the cells morphology depending on the orientation of the nanofibers [112]. Kolluru et al. focused on the tendon-to-bone insertion: they developed random mats of nanofibers of PLGA, with different degrees of mineralization (mineralized solution composed by calcium and phosphate). They found that the nanofibers morphology and mechanical properties were dependent from different degree of mineralization. The high toughness of this material was maintained without compromising the strength with the addition of hydroxyapatite mineral [83]. He et al. developed a solution for the tendon-to-bone insertion by co-electrospinning on a drum collector, a random (side) PLLGA mat of nanofibers loaded with nanohydroxyapatite (HAp), and aligned (center) PLLGA nanofibers, thus obtaining the desired gradient. They characterized the morphology and composition of the mats, addressing the gradient of alignment of the nanofibers [85]. Criscenti et al., to reproduce the ligament-to-bone interface, proposed an interesting method to combine the electrospinning and 3D printing. At first, PCL was 3D printed, obtaining a reticular structure (bone interface). Subsequently, a part of the 3D printed scaffold was covered by electrospun nanofibers of PLLGA. The nanofibers were aligned by the two collectors, producing a partial overlap on the 3D printed scaffold (enthesis). In addition, the mat of aligned nanofibers (which were not overlapped), reproduced the ligament tissue. Different mechanical properties were measured in the different parts of the scaffolds (Young’s modulus 3D print = 43.6 ± 8.1 MPa, mixed 50.6 ± 5.1 MPa electrospun = 88.9 6 ± 15.1 MPa; failure stress: 3D print = 1.62 ± 0.27 MPa; mixed = 2.57 ± 0.51 MPa; electrospun = 5.21 ± 1.11 MPa). They seeded the scaffolds with hMSCs, finding increased levels of proliferation and different orientation of the cells, depending on the side, after seven days of culture [86]. Kishan et al. to obtain patches for rotator cuff repair, developed random and aligned nanofibrous mats with a gradient of two different biodegradable BPUR10 and BPUR50. They found human BMMSCs differentiation in the different degrees of alignment of the fibers. They also reported different levels of strain depending on the disposition of the nanofibers [120]. Oliveira et al. studied the differentiation of bone marrow-derived porcine mesenchymal stem cells in ligament or bone/cartilage differentiation, using random and aligned microfibers of PCL. They added different growth factors to study the differentiation of cells in ligamentogenic, chondrogenic or fibrochondrogenic phenotype upon presentation of appropriate biochemical cues [90]. Wu et al. focused on the repair of the tendon-to-bone and ligament-to-bone insertions: they analyzed nanostructured HAp, loaded in random nanofibrous blends mats of PCL and CTS. They found increased osteoblast viability after 48 h of culture [109]. 

Zhu et al. conduced an in vivo study on a rabbit anterior cruciate ligament model, to investigate the cells growing on PLLA nanofibrous scaffolds, obtained by electrospinning on a flat plate collector with a copper grid on its surface. At the end of the animal trial, they found abundant extracellular matrix such as collagen (Types I–III) and fibrocartilage on the scaffolds [67]. Zhi et al. used random microfiber mats of SE, in culture with rabbit BMMSCs to evaluate the tendon-to-bone healing effects. After the cells test, they wrapped some mats on a resected rabbit Achilles’ tendon, and implanted the assembly in a hole of the rabbit hindlimb. After 12 weeks, they found increased bone regeneration. Moreover, the electrospun mats could not be pulled out from the bones and showed a statistically significant increment of the mechanical properties compared to the control groups [129]. 

Li et al. designed a bilayer microfibrous scaffold made of a mat of PLLA, for rotator cuff repair. A second layer of PLLA microfibers loaded with nanohydroxyapatite (HAp). The in vivo rabbit model showed that the scaffolds significantly increased the area of glycosaminoglycan staining at the tendon-to-bone interface and improved collagen organization. Implanting the bipolar membrane also induced bone formation and fibrillogenesis, as assessed by micro-CT, and histological analysis. Biomechanical testing showed that the scaffolds had a greater failure load (181.5 ± 19.0 N), failure stress (4.6 ± 0.6 MPa) and stiffness (average value > 20 MPa) than the control group at 12 weeks after surgery [66].

### 6.5. Muscle Insertions

The tendon-to-muscle interface was first addressed by Ladd et al. They co-electrospun random nanofibrous mats of a PCL and Coll blend and a PLLA and Coll blend. They obtained a gradient of the two solutions in the center of the mats and, the two distinct ones in the sides. The PCL side exhibited low Young’s modulus (4.5 ± 1.6 MPa) and failure stress (1.07 ± 0.27 MPa). This section also demonstrated the largest failure strain (130.4 ± 44.56%). In contrast, the PLLA side had the highest Young’s modulus (27.62 ± 6.06 MPa) and failure stress (3.74 ± 0.85 MPa), as well as low failure strain (35.3 ± 9.0%). The center region had the most variability in mechanical properties, but exhibited a Young’s modulus (20.1 ± 7.8 MPa) and failure stress (2.38 ± 0.60 MPa) in between the values for the PLLA side and the PCL side. The failure strain (42.8 ± 17.7%) of the central region was similar to the PLLA side. They found also an increased viability of both myoblasts and fibroblasts [68].

### 6.6. Tendon and Ligament Healing and Anti-Adhesion

Preventing inflammation and adhesion is fundamental for a successful regeneration of the tissue. Such detrimental phenomena are due to excessive proliferation of fibroblasts on the injured surface of the treated tendon or ligament. For this reason, some researchers started to study how manage the anti-adhesion problem. 

Lui et al. produced aligned nanofibers of PLLA and PCL in different percentages, charged with NPS to prevent tendon adhesion. They found that increasing the PCL content increased the failure strain but also the release rate of NPS. The failure stress was also enhanced with the addition of water as the co-solvent. This NPS-loaded scaffold showed no significant cytotoxicity, and L929 murine fibroblasts cultured on the scaffolds were able to proliferate and align along the fibers [71].

To prevent tendon adhesion, Jiang et al. investigated random nanofibrous mats of PELA loaded with the anti-inflammatory celecoxib (0%, 2%, 6%, and 10%). The mechanical tests showed a ductile behavior with different values of failure stresses depending to the percentage of celecoxib (PELA = 3.04 ± 0.32 MPa; PELA-2% = 2.87 ± 0.27 MPa; PELA-6% = 2.77 ± 0.34 MPa; PELA-10% = 2.72 ± 0.31 MPa). Cellular tests with rabbit tenocytes and dermal fibroblasts showed decreased viability, increasing the percentage of celecoxib. The in vivo rabbit model confirmed that the fibroblasts grew on the PELA mats. Moreover, the adhesions were inhibited by down-regulating the extracellular-regulated signal kinases 1/2 (ERK1/2) and small mother against decapentaplegic 2/3 (SMAD2/3) phosphorylation [72]. To promote the tendon healing and prevent adhesion, Liu et al. electrospun a random nanofibrous scaffold of PCL, as outer layer, and a blend of PCL/HA, as inner layer, to be wrapped on an injured tendon (Figure 14b). The cell viability (multipotent C3H10T1/2 cells) and in vivo chicken model, showed encouraging results in terms of anti-adhesion properties and release of HA [113]. Liu et al. electrospun random PLLA nanofibers, loaded with DGNs and bFGF, to prevent adhesions. The in vitro proliferation of the multipotent C3H10T1/2 (C3) cells showed the low affinity of these scaffolds to the cell adhesion. These results were confirmed by a rat in vivo study [69]. Zhao et al. loaded PLLA with a solution of HA and MMC, to obtain random core–shell nanofibrous mats to enhance anti-adhesion in tendon applications. The NIH/3T3 fibroblasts viability tests showed decreased cell adhesion and apoptotic effects, mediated by the release of MMC. In the in vivo rat model, the mats prevented adhesion surrounding the tendon lesion, without detrimental effect for the healing process of the injured tendon, by mediating fibroblast apoptosis and syntheses of collagen [70]. Buschmann et al. tested in vivo a DP nanofibrous random DP conduit in a rabbit Achilles tendon. After the production, the conduits were pushed-out from the tube and inverted before implantation in a rat Achilles’ tendon model. In the first study, they confirmed that DP tubes could be set around a sutured tendon rupture without any adverse effects: the cellular response of the healing tissue 12 weeks post-operation was the same as if no implant was set [125]. In the second study, the synthesis was modified to increase the elasticity. The new material was implanted in a similar rat model: the cellular response to the modified polymer, after 12 weeks, was similar to the classic DP [126]. Evrova et al. investigated a random nanofibrous bilayer tube for tendon tissue regeneration. A first layer of electrospun nanofibers of DP was electrospun on a rotating drum. A second layer of DP nanofibers, loaded with PDGF-BB, were obtained by emulsion electrospinning on the previous one (Figure 14c). The released PDGF-BB was shown to be bioactive, leading to increased proliferation of rabbit tenocytes in in vitro under serum free conditions [127]. 

To create a tendon healing and anti-adhesive scaffold, Li et al. designed a bilayer microfibrous mat, made by electrospinning first a solution of PELA and HA, and then a celecoxib-PELA solution. The in vivo data on a chicken model, confirmed that the celecoxib-loaded outer PELA layer can prevent adhesion and associated inflammation [73].

## 7. Conclusions and Future Perspective

Tendon and ligament regeneration and replacement is currently a hot topic for tissue engineering and orthopedic research. Among the various technologies explored for healing and regenerate these tissues, electrospinning is definitely one of the most promising since it combines biomimicry and manufacturing flexibility. In the last twenty years, more than one hundred scientific papers and several reviews have described different methodologies and the respective strengths and shortcomings of the electrospun nanofibers applied in the regeneration of tendons and ligaments. Researchers started investigating the effects of the nanofibers’ morphology and orientation, on the cells proliferation and growing, testing different polymeric solutions. Several in vivo tests on small and large animal models are described, providing encouraging results in terms of integration with the host tissue, healing and biocompatibility. However, despite these promising outcomes, the limited mechanical properties are currently the principal constraint in the application of these scaffolds in human clinical trials. For these reasons, the new challenge of the next years will probably be the development of electrospun multiscale hierarchical scaffolds and devices, able to replicate as soon as possible not only the hierarchical structure, but also the biomechanical properties of tendons and ligaments. Moreover, scaffolds must be customizable so that the production can be adapted to the different tendons and ligaments requiring treatment. A further enhancement will consist in personalization to meet patient-specific needs in terms of anatomy as well as resorption rate. If these results are achieved, electrospun scaffolds and devices will be industrialized and potentially become a gold standard for the surgery and the tissue engineering of tendons and ligaments.

## Figures and Tables

**Table 1:** Materials used in combination with electrospinning processes, in tendon and ligament tissue engineering.

Acronym	Extended Name	Application	References
P(LLA-CL)	Poly(l-lactide-co-ε-caprolactone)	Tendon/Ligament	[53]
		Ligament	[54]
		Tendon	[55,56]
PDLLA	Poly(d,l-lactic acid)	Ligament	[57]
PLDLA	Poly(L-lactide-co-d,l-lactic acid)	Ligament	[57]
PLLA	Poly(l-lactic acid)	Tendon/Ligament	[58,59,60,61]
		Ligament	[57]
		Tendon	[62,63,64,65,66]
		Ligament-to-Bone Interface	[67]
		Tendon-to-Muscle Interface	[68]
		Tendon Anti-Adhesion	[69,70,71]
PELA	Poly(l-lactic acid)-poly(ethylene glycol)	Tendon Anti-Adhesion	[72,73]
PDLLGA	Poly(d,l-lactide-co-glycolic acid)	Ligament	[74]
		Tendon	[75]
PLGA	Poly(lactic-co-glycolic acid)	Tendon/Ligament	[76,77]
		Ligament	[78,79,80]
		Tendon	[81,82]
		Tendon-to-Bone Interface	[83]
PLLGA	Poly(l-lactic-co-glycolic acid)	Tendon/Ligament	[84]
		Tendon-to-Bone Interface	[85,86]
		Bone-Ligament-Bone	[87]
PCL	Poly(ε-caprolactone)	Tendon/Ligament	[58,88]
		Ligament	[80,89,90,91,92,93,94]
		Tendon	[82,95,96,97,98,99,100,101,102,103,104,105,106,107,108]
		Tendon/Ligament-to-Bone Interface	[109]
		Ligament-to-Bone Interface	[110,111]
		Tendon-to-Bone Interface	[112]
		Tendon-to-Muscle Interface	[68]
		Tendon Anti-Adhesion	[71,113]
		Bone-Ligament-Bone	[87,114]
PCLDLLA	Poly(ε-caprolactone-co-d,l-lactic acid)	Ligament	[115]
PU	Poly(urethane)	Ligament	[79,116,117]
		Tendon	[118]
PEUR	Poly(ester urethane)	Ligament	[80]
PEUUR	Poly(ester urethane urea)	Tendon/Ligament	[119]
		Ligament	[74,78]
		Ligament-to-Bone Interface	[111]
PEUUR2000	Poly(ester urethane urea) elastomer	Ligament-to-Bone Interface	[110]
BPUR10	Biodegradable Poly(urethane urea) 10	Tendon-to-Bone Interface	[120]
BPUR50	Biodegradable Poly(urethane urea) 50	Tendon-to-Bone Interface	[120]
PEO	Poly(ethylene oxide)	Tendon/Ligament	[121,122]
		Tendon	[63,64,96]
PEGDA	Poly(ethylene glycol diacrylate)	Ligament	[78]
PEDOT	Poly(3,4-ethylenedioxythiophene)	Ligament	[123]
PDO	Poly(dioxanone)	Tendon	[106,124]
PAN	Poly(acrylonitrile)	Tendon	[105]
PVDF-TrFe	Poly(vinylidene fluoride-trifluoro ethylene)	Tendon	[105]
DP	Biodegradable Poly(ester urethane) block copolymer (DegraPol^®^)	Tendon Anti-Adhesion	[125,126,127]
P3HB	Poly(3-hydroxybutyrate)	Tendon/Ligament	[88]
Nylon6.6	Nylon 6.6	Tendon/Ligament	[60]
SE	Silk	Ligament	[123]
		Tendon	[128]
		Tendon-to-Bone Interface	[129]
SF	Silk Fibroin	Tendon	[56]
Fibrinogen	Fibrinogen	Tendon/Ligament	[121]
		Tendon/Ligament	[59,60]
Coll	Collagen	Tendon	[55,62,118]
		Tendon-to-Muscle Interface	[68]
CTS	Chitosan	Tendon/Ligament-to-Bone Interface	[109]
		Tendon	[63,97,104,108]
		Tendon Anti-Adhesion	[113]
GT	Gelatin	Tendon	[63]
HA	Hyaluronic acid	Tendon Anti-Adhesion	[70,73,113]
mGLT	Methacrylated Gelatin	Tendon	[100]
Carbothane™ 3575A	Poly(carbonate)-based thermoplastic poly(urethane)	Tendon/Ligament	[130]
MWCNTs	Multi Wallen Carbon Nanotubes	Tendon/Ligament	[130]

**Table 2:** Electrospun blends and core–shell fibers and their applications in tendon and ligament tissue engineering.

Acronym	Type	Application	References
P(LLA-CL)/Coll	Blend	Tendon	[55]
P(LLA-CL)/SF	Blend	Tendon	[56]
PLLA/PCL	Blend	Tendon Anti-Adhesion	[71]
PLLA/MMC	Core–Shell	Tendon Anti-Adhesion	[70]
PLLA/Coll	Blend	Tendon/Ligament	[59,60]
	Core–Shell	Tendon	[62]
	Blend	Tendon-to-Muscle Interface	[68]
PLLA/PEO	Blend	Tendon	[64]
PLLA/PEO/CTS/GT	Blend	Tendon	[63]
PEO/Fibrinogen	Blend	Tendon/Ligament	[121]
PCL/CTS	Blend	Tendon/Ligament-to-Bone Interface	[109]
		Tendon	[104,108]
PCL/Coll	Blend	Tendon-to-Muscle Interface	[68]
PCL/HA	Blend	Tendon Anti-Adhesion	[113]
PCL/PLGA	Blend	Tendon	[82]
PLGA/Coll	Blend	Ligament	[79]
PLGA/PEGDA	Blend	Ligament	[78]
PEUUR/PEGDA	Blend	Ligament	[78]
PEUUR/PCL	Blend	Ligament	[80]
PU/Coll	Blend	Tendon	[118]
PELA/HA	Blend	Tendon Anti-Adhesion	[73]

**Table 3:** Particles and drugs to load electrospun fibers and their applications in tendon and ligament tissue engineering.

Acronym	Extended Name	Application	References
bFGF	Basic Fibroblast Growth Factor	Tendon/Ligament	[76,84]
		Tendon Anti-Adhesion	[69]
		Tendon	[81]
DGNs	Dextran Glassy Nanoparticles	Tendon Anti-Adhesion	[69]
Celecoxib	Selective Non-Steroidal Anti-Inflammatory Drug	Tendon Anti-Adhesion	[72,73]
MMC	Mitomycin-C	Tendon Anti-Adhesion	[70]
TSA	Trichostatin-A	Tendon	[64]
HAp	Hydroxyapatite	Tendon/Ligament-to-Bone Interface	[109]
		Tendon-to-Bone Interface	[85]
		Ligament-to-Bone Interface	[110,111]
		Tendon	[66]
CNCs	Cellulose Nanocrystals	Tendon	[104,108]
CTGF	Connective Tissue Growth Factors	Ligament	[93]
PDGF-BB	Platelet Derived Growth Factor-BB	Tendon	[127]
TP	Tricalcium Phosphate	Tendon-to-Bone Interface	[83,131]
BLM	Biomimetically Prepared Bone-like Mineral	Bone-Ligament-Bone	[114]
